# Chronic Lymphocytic Leukemia B-Cell Normal Cellular Counterpart: Clues From a Functional Perspective

**DOI:** 10.3389/fimmu.2018.00683

**Published:** 2018-04-04

**Authors:** Walaa Darwiche, Brigitte Gubler, Jean-Pierre Marolleau, Hussein Ghamlouch

**Affiliations:** ^1^EA 4666 Lymphocyte Normal – Pathologique et Cancers, HEMATIM, Université de Picardie Jules Verne, Amiens, France; ^2^Laboratoire d’Hématologie, Centre Hospitalier Universitaire Amiens-Picardie, Amiens, France; ^3^Laboratoire d’Oncobiologie Moléculaire, Centre Hospitalier Universitaire Amiens-Picardie, Amiens, France; ^4^Service d’Hématologie Clinique et Thérapie cellulaire, Centre Hospitalier Universitaire Amiens-Picardie, Amiens, France; ^5^Institut National de la Santé et de la Recherche Médicale (INSERM) U1170, Gustave Roussy, Villejuif, France; ^6^Institut Gustave Roussy, Villejuif, France; ^7^Université Paris-Sud, Faculté de Médecine, Le Kremlin-Bicêtre, France

**Keywords:** chronic lymphocytic leukemia B-cell, chronic lymphocytic leukemia, B-cell subsets, B-cell differentiation, normal cellular counterpart, transitional B cell, memory B-cell, antibody-secreting plasma cell

## Abstract

Chronic lymphocytic leukemia (CLL) is characterized by the clonal expansion of small mature-looking CD19+ CD23+ CD5+ B-cells that accumulate in the blood, bone marrow, and lymphoid organs. To date, no consensus has been reached concerning the normal cellular counterpart of CLL B-cells and several B-cell types have been proposed. CLL B-cells have remarkable phenotypic and gene expression profile homogeneity. In recent years, the molecular and cellular biology of CLL has been enriched by seminal insights that are leading to a better understanding of the natural history of the disease. Immunophenotypic and molecular approaches (including immunoglobulin heavy-chain variable gene mutational status, transcriptional and epigenetic profiling) comparing the normal B-cell subset and CLL B-cells provide some new insights into the normal cellular counterpart. Functional characteristics (including activation requirements and propensity for plasma cell differentiation) of CLL B-cells have now been investigated for 50 years. B-cell subsets differ substantially in terms of their functional features. Analysis of shared functional characteristics may reveal similarities between normal B-cell subsets and CLL B-cells, allowing speculative assignment of a normal cellular counterpart for CLL B-cells. In this review, we summarize current data regarding peripheral B-cell differentiation and human B-cell subsets and suggest possibilities for a normal cellular counterpart based on the functional characteristics of CLL B-cells. However, a definitive normal cellular counterpart cannot be attributed on the basis of the available data. We discuss the functional characteristics required for a cell to be logically considered to be the normal counterpart of CLL B-cells.

## Introduction

B-cell chronic lymphocytic leukemia (CLL) is characterized by clonal proliferation and accumulation of mature CD5+ B lymphocytes in bone marrow, peripheral blood, and lymphoid tissues (1, 2). Despite the homogeneous morphology, transcriptional profile, and immunophenotype, CLL is clinically a heterogeneous disease where some patients never require therapy and some patients display an aggressive course with poor response to therapy. CLL can be divided into two groups based on the immunoglobulin heavy-chain variable gene (IGHV) mutational status that have significantly disparate clinical outcomes with mutated IGHV cases have significantly superior outcomes compared to unmutated ones. Cytogenetic aberrations including 17p deletion, 11q deletion, trisomy 12, and 13q deletion have been associated with prognosis in CLL (1, 3). The genetic landscape of CLL showed a marked inter-patient genetic heterogeneity together with complex clonal organization and epigenetic status (2, 3). The vast majority of CLL patients exhibit a precursor state, known as monoclonal B-cell lymphocytosis (MBL). The current advances on CLL molecular pathogenesis, genetic and epigenetic features, clinical presentation, and treatment are excellently reviewed in Ref. (1–3).

In hematologic malignancies, determination of the cell-of-origin (the cell in which the first oncogenic event occurred) and the normal counterpart of malignant cells (the cell in which the final transformation occurred) is important to elucidate the pathogenesis, mechanisms, and natural history of the disease with implications for treatment. Malignant lymphocytes are considered to maintain the key features (e.g., phenotype or differentiation program) of the differentiation stage of their normal cellular counterpart (4, 5). The normal counterpart of malignant B-cells in CLL remains controversial despite investigation by various approaches. Studies based on immunophenotypic, IGHV mutational status analysis, gene expression profiling [reviewed in Ref. (6–8)], microRNAome (9), lncRNA expression (10), and, very recently, epigenetics (11–13) have tried to demonstrate similarities between CLL B-cells and normal B-cells isolated *ex vivo*. However, few studies have taken functional characteristics into account to address the issue of the normal counterpart of CLL B-cells (14, 15). B-cell subsets differ substantially in terms of their activation requirements, functional capacities, and requirements and propensity for plasma cell (PC) differentiation. *In vitro* B-cell activation by T-dependent or T-independent stimuli can be used to measure the proliferation and differentiation potential of the B-cell subsets (16). Activation and differentiation requirements may reveal intrinsic differences or similarities between normal B-cell subsets and malignant B-cells. Several studies have assessed the activation and differentiation capacity of CLL B-cells *in vitro* and *in vivo* and have shown that these cells are able to differentiate into antibody-secreting plasma cells (ASPCs) with specific requirements (14, 17–24). This review discusses the normal counterpart of CLL B-cells from a functional perspective. The first section of this review summarizes the current data regarding peripheral B-cell differentiation and human B-cell subsets. The following section will try to define the subset(s) of human B-cells with similar activation and terminal differentiation requirements to those of CLL B-cells.

## B-Cell Subsets and Terminal Differentiation

### Peripheral B-Cell Development

**Figure 1:**
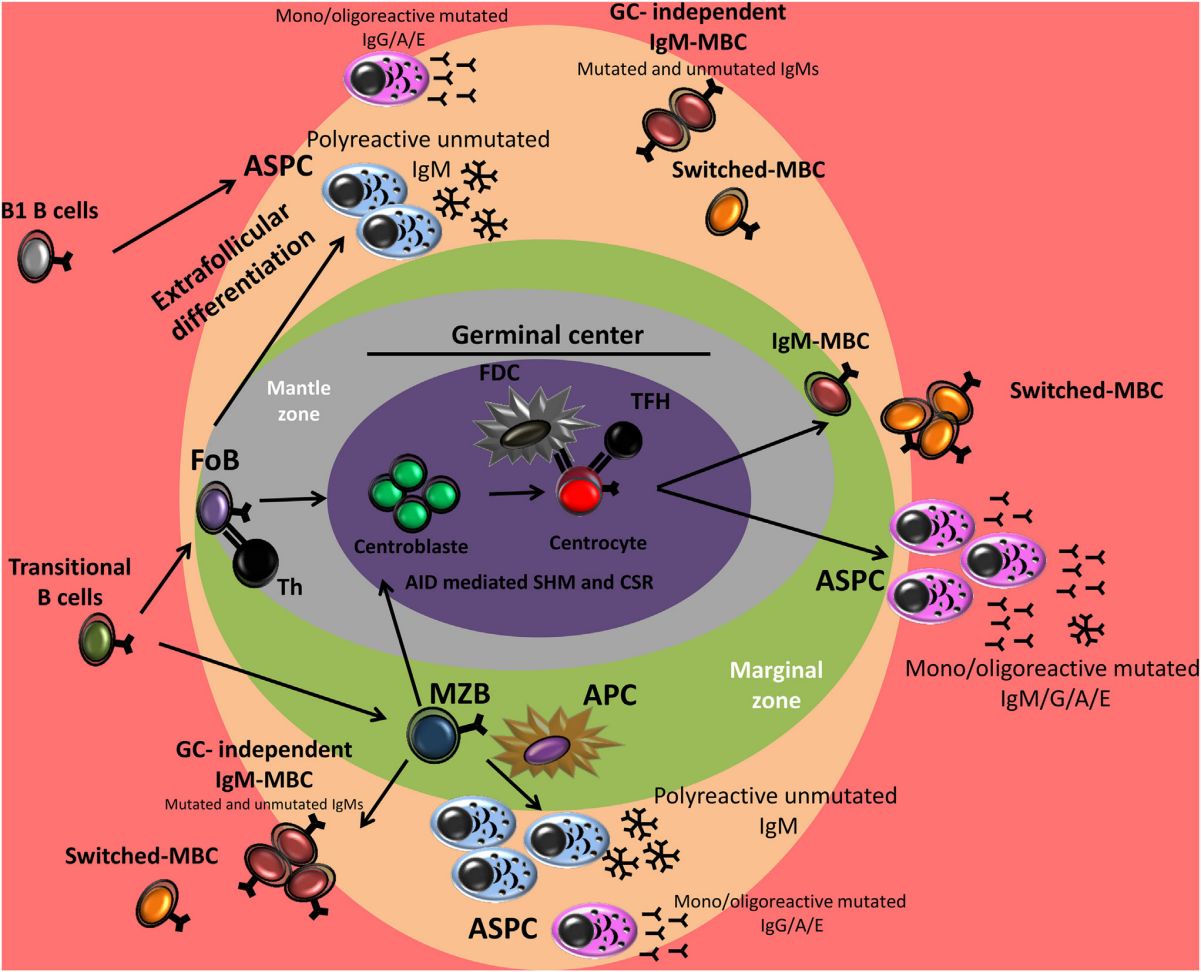
B cell differentiation in germinal center (GC)-dependent and extrafollicular pathways. After antigen encounter, activated marginal zone B cell (MZB) and activated follicular B cell (FoB) may follow two pathways: (i) extrafollicular differentiation into plasma cells (PCs) but also memory B-cells (MBCs) formation independently of the GC reaction or (ii) GC formation in which the B cells can undergo somatic hypermutation (SHM) and/or class switch recombination (CSR) and become a high-affinity MBC or a PC secreting high-affinity antibodies. In mice, B1 cells respond to T cell-independent antigens and generate predominantly low-affinity IgM or isotype-switched PCs. The contribution of B1 cells to the MBC compartment is recently identified. Th, T helper cell; FDC, follicular dendritic cell; TFH, T follicular helper cell; APC, antigen-presenting cell.

B-cell subsets have been identified and subdivided on the basis of their development, phenotype, location, and functional differences that reflect their different phenotypes. The vast majority of studies characterizing B lymphocyte development and function have been performed on mice, but recent data have highlighted significant differences between murine and human B-cell development [reviewed in Ref. (25, 26)]. In human and in mice, mature B-cell development takes place first in the bone marrow from hematopoietic stem cells (HSCs) to immature B-cells, then in the periphery from transitional to fully mature B-cells. During early B-cell differentiation in the bone marrow, functional recombination of V, D, and J segments in pro- and pre-B-cells allows the cells to develop into immature B-cell that express surface IgM. Bone marrow immature B-cells start to express surface IgD to complete their maturation into fully mature naive B-cells. Surface IgD promotes B-cell survival and attenuates anergic B-cell responses to self-antigen (27). B-cells between the stages of immature B-cells and fully mature naive B-cells are called transitional B-cells. Transitional B-cells emigrate to peripheral lymphoid organs [spleen, lymph node, and mucosa-associated lymphoid tissues (MALT)] *via* peripheral blood, where they account for 5–10% of all B-cells (28). Once in peripheral lymphoid organ tissue, transitional B-cells rapidly pass through transitional phases before committing to either naive follicular (Fo)B-cells or marginal zone (MZ)B-cells (29). The fate of cells to develop into either FoB-cells or MZB-cells depends on several signaling pathways, including the B-cell receptor (BCR), NOTCH2, B-cell-activating factor (BAFF) receptor, and the canonical nuclear factor-kappaB pathway, as well as signals involved in the migration and anatomical retention of MZB-cells (29). Naive B-cells recirculate between peripheral blood (where they represent about 65% of all B-cells) and lymphoid tissues and, if they encounter antigens (Ags), they differentiate into Ag-experienced memory B-cells (MBCs) or PCs (Figure 1). Naive B-cells die after several days if they do not encounter any Ags.

### B-Cell Subsets and Function

Transitional B-cells are thought to be functionally immature and have a characteristic phenotype, which includes expression of surface membrane IgM and IgD, CD21, CD22, CD5, and high expression levels of CD24 and CD38 (29). The transitional stage consists of cells at different stages of maturation between the immature and naive mature B-cell compartments. They, therefore, typically display heterogeneous features; these cells have unmutated IGHV genes and present different capacities to proliferate and differentiate into ASPC after *in vitro* stimulation compared to other B-cell subsets (9, 28, 30–32). In human, maturation into CD5+ pre-naive B-cells is accompanied by downregulation of CD38 and CD24, making them partially responsive to BCR stimulation and CD40 ligation (CD40L) (30). Pre-naive B-cells downregulate CD5 expression and become naive B-cells, which are fully responsive to antigen (30).

Naive follicular B-cells mainly reside in B-cell follicles in the white pulp of the spleen and in the cortex of lymph nodes and are found in other organized lymphoid tissues such as Peyer’s patches and tonsils (29). Naive FoB-cells recirculate between peripheral lymphoid tissues until they encounter their specific Ag. These B-cells are typically involved in the response to T-dependent (TD) Ags *via* the formation of germinal centers (GCs) leading to the production of ASPCs, but also MBCs. However, they are also capable of responding to T-independent (TI) Ags (33, 34).

Marginal zone B-cells are generated as naive B-cells. However, they have a pre-activated phenotype and the ability to self-renew, resembling those of memory cells (29). In mice, MZB-cells are restricted to the MZ of the spleen. In humans, MZB-cells are also found in the inner wall of the subcapsular sinus of lymph nodes, the epithelium of tonsillar crypts, and the subepithelial area of MALT, including the subepithelial dome of intestinal Peyer’s patches (29). Splenic MZB-cells in humans play an important role in TI immune responses to blood-borne Ags and are responsible for systemic immune responses to bacterial polysaccharide Ags (29, 35). Humans without a functional spleen are vulnerable to infections by encapsulated bacteria due to their inability to form protective MZB-cell-derived antibody responses against TI Ags (36). MZB-cells also participate in TD immune responses. MZB-cells capture, process and present Ags, and deliver costimulatory signals to T-cells more rapidly and more efficiently than FoB-cells both *in vitro* and *in vivo* (29). MZB-cells have been ascribed other functions, including production of “natural” IgM in the absence of an immune response (29, 35). Although they share some functional and phenotypic characteristics with their murine counterpart, human MZB-cells possess distinct characteristics, including the ability to recirculate through lymphoid organs and the presence of somatic mutations of IGHV genes (37, 38). These differences with murine MZB are the reason why human MZB are considered to be MBCs, as a link between human blood CD27+ IgM+ IgD+ cells [hereafter called IgM memory B-cells (IgM MBC)] and MZB-cells in the spleen has been proposed on the basis of the marked similarly between the two cell types (39–41). The existence of an MZ cell lineage in humans remains a subject of debate.

In mice, B-cells are mainly subdivided into B2 cells, including MZB-cells and FoB-cells, and B1 cells. B1 cells can be further subdivided into CD5+ B1a and CD5− B1b subsets. Murine B1 cells are mostly found in the peritoneal cavity, but a few B1 cells also reside in the spleen and lymph nodes (42). In non-human primates, a B1-like B-cell population has been identified in serosal cavities that exhibit phenotypic and functional similarities to murine B1 cells (43). In humans, CD5 is expressed on up to 60–75% of B cells of umbilical cord blood. A decrease is seen in the peripheral blood of adults where human CD5+ B cells represent about 3% (1–8%) of peripheral blood lymphocytes and 5–30% of the circulating B cells (44, 45). In addition to transitional T1 B-cells, CD5 is also expressed in a subset of regulatory B-cells (Bregs) and in a minor subset of pre-naïve, naïve mature, and MBCs (28, 32, 46–48). In humans, the presence of B1 B-cell subsets remains controversial. However, Rothstein’s team has identified a subset of B-cells in umbilical cord and adult peripheral blood that express CD20, CD27, and CD43 as human B1 cells (47, 49). The authors found that these cells also express CD5 (75% are CD5+), although they represented only a minority (34%) of CD20+ CD5+ B-cells in adult peripheral blood. These B1-like cells display functional characteristics associated with murine B1 cells, including efficient T-cell stimulation and efficient antigen presentation, tonic intracellular signaling, spontaneous secretion of IgM, and expression of unmutated antibody genes. However, the identification and role of these cells have been questioned by several groups (50, 51). Remarkably, the phenotype and transcriptional profile (49) of the human B1-like cells subset partly overlaps with that of circulating human CD27+ IgM+ IgD+ B-cells (IgM MBCs). Due to their predominance during fetal ontogeny and neonatal life, B1 B-cells are thought to act as a first line of defense against invading pathogens that can be neutralized by the polyreactive IgM secreted by these cells (43, 52). B1 cells respond to TI Ags and rapidly form plasmablasts that proliferate in extrafollicular foci in the spleen and can give rise to PCs in the spleen, omentum, and lamina propria of the gut (53). In addition to generating natural antibodies (Abs), B1 B-cells also actively contribute to antigen-induced immune responses (e.g., Ags from *Francisella* spp., *Borrelia hermsii, Salmonella typhi, Streptococcus pneumoniae*, and influenza virus) and can generate TI- and GC-independent antigen-specific memory/ASPC, ensuring a long-lasting immune response (54–56).

A minor fraction of normal B-cells exerts regulatory functions and produces immunosuppressive factors, such as interleukin 10 (IL-10), and are called Bregs—found within the CD19+ CD24hiCD38hi immature B-cell subpopulation—or IL-10-producing B-cells (B10)—found within the CD19+ CD24hiCD27+ B-cell subpopulation (48). These Bregs play an important role in regulating innate and adaptive immune responses during inflammation, autoimmunity, and cancer (48). B10 cell development and function appear to be predominantly driven by antigen-receptor signals (both innate and adaptive immune signals). CD40 activation is the best characterized signal known to induce differentiation of CD24hiCD38hi Bregs. A CD5+ CD24hiCD27+ B10 progenitor for human B10 cells was identified and can differentiate into functional B10 cells following costimulation with toll-like receptor (TLR) ligand (LPS and CpG) (48). A functional link between B10 and anergic B-cells has been established, as both populations arise following chronic exposure to antigen and express low levels of surface immunoglobulin M (sIgM) (48). A small fraction of B10 can differentiate into polyreactive and/or Ag-specific Ab-secreting PCs after terminating IL-10 production *in vivo* and *in vitro* (57). However, the possible regulatory role played by these Abs has yet to be investigated.

### Memory B-cell Subsets

Memory B-cells mediate the secondary humoral immune responses. During these responses, B-cell activation, proliferation, and differentiation are faster with the secretion of higher affinity Abs compared to primary responses (33, 58). These qualitative and quantitative differences between primary and secondary antibody responses are due to the increased frequency and affinity of Ag-specific B-cells and the intrinsic differences between memory and naive B-cells. MBCs can survive for several months in the absence of antigenic stimulation and provide an early antibody response against recurrent infections (59). In humans, up to 40% of B-cells in peripheral blood are MBCs and can be subdivided into separate pools based on IgM, IgD, and CD27 expression. MBCs exist in two main types: (i) immunoglobulin (Ig)-switched MBCs (CD27−/CD27+ IgD− IgG/A/E+) and (ii) unswitched MBCs expressing IgM, which include IgM-only MBCs (CD27+ IgM+ IgD−) and IgM MBCs (CD27+ IgM+ IgD+, IgM MBC), each of which accounts for about 15–20% of total B-cells (37, 39, 41). IgM MBC share several functions and phenotypic characteristics with human B1-like cells, MZB-cells, and mouse B1a cells (49, 60–62); they are thought to be the major source of “natural” Abs in the body, can express Igs with low-frequency somatic hypermutation (SHM) and produce IgM (but also some IgG and IgA, after *in vitro* differentiation) (60, 61). High-throughput Ig VHDJH sequencing of human B-cell subsets showed that IgM-only subsets are related to CD27+-switched MBCs and are GC-derived MBCs (41). Transcriptional expression profiling showed that human IgM+ MBCs are more similar to IgG+ MBCs than to naive B-cells, but with distinct functional capacities (38). Indeed, following secondary challenge with antigen, unswitched MBCs have been shown to preferentially enter GCs and therefore play an active role in sustaining memory, while switched MBCs preferentially form plasmablasts (38, 63, 64).

With aging, certain viral infections and autoimmune diseases, B-cell subsets with distinctive phenotypic and functional features were identified. In mice, an age-associated B-cell (ABC) population presenting a characteristic transcriptional profile and features of Ag-experienced cells was described (65). Phenotypically, these CD19+ B-cells are negative for CD21 and CD23, express CD11c and intermediate level of CD5 and are IgM+. An ABC-like (representing between 0.8 and 4% of circulating B-cells) was identified in human blood of elderly healthy subjects. These cells express low levels of CD23 and high levels of CD27 and CD5 but unlike in mice, human ABC-like are isotype switched (65). However, in both mice and humans, they express and are characterized by a T-bet driven transcriptional program and appear to arise and expand in the context of autoimmune disease, parasite infections, and viral infections. Following TLR9 or TLR7 stimulation, murine ABCs robustly proliferate, rapidly differentiate into ASPC that secret switched Abs (IgG2a/c) and produce regulatory cytokines such as IL-10 and interferon (IFN)-γ, however, they respond poorly to BCR or CD40 stimulation (65). Given their functional attributes, Ag-experienced profile and atypical activation state, age-associated B cell were proposed to represent an MBC subset generated during response to nucleic acid-containing Ags in the presence of inflammatory cytokines (65). It is important to highlight here that a subset of human T-bet+ CD11c+ CD21− MBC called atypical MBCs (or exhausted MBCs) was also described to expand in viral (ex. HIV and HCV) and parasitical infections (up to 50% of circulating B cells) and to be enriched among IgG1+ and IgG3+ B-cells (66, 67). These atypical MBCs present distinct functional features with mice ABCs (as they do not proliferate or differentiate or produce IL-10 and IFN-γ in response to TLR agonists) questioning the possible relationship between these subsets (67).

### Memory B-cells Can Also Be Generated by a GC-Independent Pathway

Memory B-cell subsets present different frequencies of somatic mutation and various replication histories that are considered to reflect their generation in primary or secondary GCs. Nevertheless, MBCs and memory-like B-cells can be generated in responses not necessarily involving GC formation and IGHV SHM (e.g., extrafollicullar and TI responses) (Figure 1) (60, 62, 68, 69). Moreover, in mice, recent data distinctly show that B1 cells (B1a and B1b) can also generate MBCs during TI immune responses (54–56, 62).

While the GC origin of switched MBCs and IgM-only MBCs is generally accepted, the origin of IgM+ IgD+ CD27+ MBCs remains disputed (37, 41, 70, 71). A GC-independent origin of these cells is supported by the presence of IGHV somatic mutations in patients with hyper-IgM syndrome type I (characterized by CD40L gene mutations) and in IgM+ IgD+ CD27+ cells from cord blood. It has recently been proposed that the majority of CD27+ IgM+ IgD+ B-cells are generated by a GC reaction [the pros and cons of GC origin of these cells are discussed in Ref. (37, 71)]. A very recent study designed to characterize MBC development in children of different ages (the study involved asplenic children) showed that three types of IgM MBCs can be distinguished with different developmental histories: (i) innate IgM MBCs, the largest pool in infants, are generated in the spleen by a GC-independent mechanism; (ii) remodeled innate IgM MBCs that participate in the GC reaction and accumulate somatic mutations; and (iii) IgM MBC newly produced by the GC reaction (72). The authors concluded that most IgM MBCs have a GC-independent origin, but with age they become remodeled in the GC. These data are in line with a previous work that identified a subset of B-cells in human infants that undergoes repertoire diversification *via* antigen-independent VH gene SHM (73). The generation and features of MBCs in humans are elegantly reviewed by Seifert and Kuppers et al. (37) and in humans and mice by Weisel and Shlomchik (25).

### B-Cell Subsets and BCR Reactivity

As B-cells develop and differentiate, they go through different stages of random gene rearrangement and SHM, inevitably leading to the production of B-cells expressing autoreactive BCR. To prevent the potential development of autoimmunity, autoreactive B-cells are eliminated at early stages of B cell development in the bone marrow (central tolerance) and at later stages in the peripheral lymphoid organs (peripheral tolerance). Tolerance mechanisms include clonal deletion, receptor editing, or anergy. However, about 50% of immature and transitional B-cells present an autoreactive BCR and 7% of these B-cells express a polyreactive BCR (74, 75). The percentage of clones with autoreactive BCRs decreases to 20%, while clones with polyreactive BCR decrease to 4% through maturation into naive B-cells (74, 75). The percentage of autoreactive BCR among IgM+ MBCs drops to 2%, suggesting a selection checkpoint against autoreactivity during IgM+ MBC development in humans and that naive B-cells expressing autoreactive Abs do not contribute to the IgM+ MBC compartment (75, 76). By contrast, BCR auto- and polyreactivity is increased in IgG-memory cells to 30 and 23%, respectively (76, 77), and this is linked to SHM activity or to a failure of GC exclusion of self-reactive B cells (76). However, in terminally differentiated bone marrow IgG-positive PCs, the frequency of autoreactive BCR range from 2 to 27% and that of polyreactive BCR decreases to 10%, suggesting selection against secreted auto- and polyreactive Abs in switched-PC compartment (78). It has been suggested that multiple rounds of GC selection leading to the formation of bone marrow PCs lead to higher loads of SHM, and high specificity may be the end product of iterative affinity maturation processes.

### Terminal Human B-Cell Differentiation Into Antibody-Secreting Plasma Cell

Plasmablasts and PCs are terminally differentiated cells of the B-cell lineage that secrete high levels of Abs. However, B-cell subsets differ in terms of their location, ability to migrate, response to TI- or TD-Ag, and the stimulation requirements and propensity for ASPC differentiation (79).

B-cells can respond to TI-Ags that either activates them *via* BCR and innate receptors such as TLR (TI type 1 Ag) or *via* extensive crosslinking of BCR due to the repetitive nature of the Ag (TI type 2 Ag) (80, 81). TI responses are usually directed against blood-borne pathogens in the splenic MZ and in mucosal tissues, where B-cells proliferate and rapidly differentiate into plasmablasts and PCs in extrafollicular areas (40). In the context of TI responses, isotype switching and affinity maturation are limited and result in the formation of short-lived PCs that predominantly produce low-affinity, polyreactive IgM (33, 79).

The production of ASPCs in response to TD-Ags occurs in two sequential overlapping responses, with the first response called “extrafollicular response” leading to immediate protection, while the second response provides persistent protection known as the “follicular response” (79). The segregation of B-cells between these two responses is mediated by the G protein-coupled receptors, Epstein–Barr virus induced molecule-2 (also known as GPR183) (82) and signaling lipid sphingosine-1-phosphate (83).

In the extrafollicular response, B-cells migrate to the splenic bridging channels or junction zones at the border between T zones and red pulp or lymph node extramedullary cords, and then rapidly proliferate and differentiate into early short-lived (3–5 days) plasmablasts that are a major source of germline polyreactive IgM Abs (33, 34), although class switch recombinations (CSRs) and small SHMs can occur (34, 58, 84–86). Recent data suggest an important role for Bcl6+ PD1low pre-GC T follicular helper (TFH) in the priming of this response (87, 88). This pathway is important for early protection against microbial infections and is observed in responses to many pathogens, including *Ehrlichia muris, Borrelia burgdorferi*, and *Salmonella typhimurium* (84). Although long-lived PCs are thought to be generated by GC reaction, recent data show that long-lived IgM-secreting PCs can be generated in a GC-independent extrafollicular manner (85, 89, 90). These long-lived IgM-secreting PCs accumulate somatic mutations in their IgV locus in an activation-induced cytidine deaminase (AID)-induced manner (85).

In the follicular pathway, activated B-cells form the GC, where they proliferate and clonally expand under the influence of TFH cells and follicular dendritic cells (33, 34). In the GC, B-cells continue to rapidly proliferate and they undergo CSR to antibody classes other than IgM, acquire SHMs of V regions and are selected on the basis of antigen affinity (33). B-cells leave the GC as plasmablasts and long-lived PCs that are capable of sustaining a high level of high affinity antibody secretion or as memory cells (33). It has been proposed that the early phases of GC reactions can give rise to IgM+ IgD+ and a few IgM-only or class-switched MBCs. However, most MBCs generated during late stages of GC reactions are class-switched (37). For example, this pathway is observed in responses to influenza (91).

Human ASPCs are heterogeneous (79, 92). This heterogeneity is determined by the type of stimuli (Antigen T-independent/T-dependent antigen, cytokines, and partner cells), the exact anatomical site (lymph nodes, spleen, gut, tonsil, and bone marrow) and, most importantly, the B-cell type (naive, classical memory, IgM memory, MZ, or B1-like B-cells) (71, 92–95). The propensity of a B-cell to differentiate into a PC is also the result of the extent to which it has been developmentally pre-programmed to differentiate. An example of the impact of anatomical site is the higher frequency of IGHV gene mutation and CSR to IgA in MBCs from MALT compared to memory cells in other lymphoid tissues, such as lymph nodes, in which IgG is typically predominant (71).

## *In vitro* Modeling of Human Terminal B-Cell Differentiation

*In vitro* studies of human terminal B-cell differentiation have contributed to the discovery that B-cell subsets have distinct activation requirements and differ in terms of their responsiveness to stimulating agents, the intensity of the response and the propensity to undergo PC differentiation, SHM, and isotype switching (79).

Much of the cellular and molecular findings concerning terminal B-cell differentiation into ASPCs in humans are derived from *in vitro* models. Several aspects of ASPC differentiation can be effectively reproduced *in vitro*. Progress in our knowledge about the biology of human B-cell and ASPC differentiation has led to the development of new *in vitro* differentiation models taking into account the B-cell type as well as the nature of the antigen and the costimulatory signals and cytokines that determine the broad features of the resulting PCs (96–98). In humans, the majority of these studies are performed by using peripheral blood B-cells (16, 31, 38, 59, 99–121) as a source of naive circulating FoB-cells, switched and IgM MBCs, transitional B-cells, and mature CD5+ B-cells. However, various studies have been performed using tonsillar (71, 95, 122–124), splenic B-cells (125–127), or cord blood B-cells (as a richer source of transitional B-cells) (31, 111, 121, 124).

### Different B-Cell Types: Different Activation and Differentiation Requirements

B-cell subsets present distinct potentials for differentiation into ASPCs. Earlier studies highlighted differences in response between human B-cell subsets using density fractioning or fluorescent activated cell sorting (FACS). For example, Suzuki and Sakane found that normal peripheral blood B-cells sedimenting in a high density fraction on a Percoll density gradient consist of small resting B-cells, while Percoll-separated low-density B-cells correspond to activated large B-cells (128). Stimulation of small resting B-cells (showing high density on Percoll) by *Staphylococcus aureus* Cowan I (SAC) (which cross-links the BCR) induces vigorous proliferation with no subsequent differentiation into ASPCs, while activated large B-cells (showing low density on Percoll) differentiate directly into ASPCs without the need for extensive proliferation (128). Subsequent studies using FACS-sorted surface lgD+ (naive) and lgD− (memory) B-cells activated by SAC revealed that these populations exhibit differences in their differentiation outcome (129), as naive and MBCs differ in terms of their *in vitro* responsiveness to stimulation, mimicking primary and secondary responses *in vivo*. It is now well known that MBCs respond more rapidly and more vigorously to antigenic stimulation than naive cells (38, 59, 100, 125, 127, 130).

Naive and MBCs present differences in gene expression that could explain their differences in response to stimulation and subset-specific cell-intrinsic features play an important role in their terminal differentiation (38, 131, 132). Gene expression profiles (GEPs) of CD27+ MBCs (IgG+ CD27+ and IgM+ CD27+ B-cells) differ from those of naive B-cells and are enriched in gene signatures that are associated with enhanced antigen responsiveness and plasmablast differentiation (38, 132). MBCs present higher expression of cell surface receptors and costimulatory molecules including TLRs (TLR7/9/10), CD21, CD27, CD80, CD86, CD122, and TACI (105, 114, 132, 133). Moreover, MBCs appear to be more metabolically active than naive B-cells (62). MBCs compared to naive B-cells, express lower levels of transcription factors that are important in maintaining cellular quiescence, such as promyelocytic leukemia zinc finger factor and Krüppel-like factors (KLF)4 and KLF9 (131, 132). Furthermore, ASPC differentiation from MBCs requires less STAT3 function than generation of ASPC from naive B-cells (131). Recent data show that, following multivalent BCR crosslinking, the magnitude of activation of downstream components of the BCR signaling pathway (e.g., phosphorylation of S6 ribosomal protein and IKBalpha degradation) was greater in MBCs than in naive B-cells (127). These phenotypic and molecular characteristic of naive and MBCs reflect the functional properties of these B-cell populations.

### Behavior of Naive and MBCs in the Presence of CpG Oligodeoxynucleotide (ODN) and CD40L

Human naive and MBCs can both be induced to become terminally differentiated ASPC in response to CD40L and cytokines (called the CD40 system) or to bystander help, but with substantial differences in terms of proliferation, differentiation capacity, and isotype switch (16, 99, 101, 102, 122, 125, 126, 134–136). The CD40 system induces a few number of naive B-cells to differentiate into IgM-secreting PCs (few of which go on to produce IgG and IgA) (16, 99, 102–104, 123, 125, 135). Under the same stimulation conditions, most CD27+ MBCs predominantly differentiate into IgG-secreting ASPCs (38, 59, 99, 102, 104–107, 123, 125, 135).

In humans, naive B-cells are minimally responsive to CpG ODNs, which are a TLR9 ligand, but MBCs proliferate and differentiate into ASPCs (16, 108–114). Human naive CD27− B-cells express very low levels of TLRs and need to be stimulated *via* their BCR or by IFN-α (116) to express TLR9 and become responsive to CpG stimulation (105, 114, 115). Some studies have shown that a specific culture system using CpG together with a combination of BCR engagement and T-cell help *via* CD40 signaling can also induce plasma-cell differentiation of naive human B-cells that predominantly produce IgG, but also some IgM and IgA (115, 117, 118). However, one group has described human naive B-cell activation and differentiation into IgM-secreting PCs by the use of TLR9-activating ODN alone, but with only low-level IgM secretion (119). In addition to TLR9, human naive and MBCs respond to TLR7 agonist by proliferating and differentiating into IgM- and IgG-producing cells in the absence of BCR stimulation and CD40–CD40L interaction (120).

Transitional B-cells are thought to be functionally immature naive B-cells; they coexpress IgM and IgD and have unmutated Ig variable regions. CD24bright CD38+ transitional human B-cells (isolated from peripheral blood or cord blood) have been characterized as the main non-MBC subset responsive to TLR9 activation (111, 124). In response to TLR9 stimulation, these cells upregulate the expression of AID and BLIMP-1, differentiate into ASPCs primarily producing polyreactive “natural” and anti-polysaccharide IgM, but also some IgG (31, 111, 121, 124, 137, 138). AID expression by human and murine transitional B-cells has been shown to be essential for central B-cell tolerance and to remove autoreactive clones *via* its recombination-activating gene (RAG)-coupled genotoxic activity (137, 138). In response to CpG, human transitional B-cells have also been shown to generate somatically mutated IgM+ IgD+ CD27+ MBCs (139). These findings are further supported by studies indicating that engagement of TLR4 or TLR9 in murine transitional 1 B-cells promotes CSR and the development of ASPCs (140, 141).

Because of their distinct phenotypes, it has been suggested that human CD5+ and CD5− B-cells may have different activation requirements (142, 143). The earliest studies were unable to show different responses to stimuli between CD5+ and CD5− B-cells because of the purity of isolated CD5+ B-cells (50–90%) (143–145). However, different responses following activation by surface Ig ligands and cytokines have been observed by using highly purified peripheral blood CD5+ and CD5− B-cells by FACS sorting (142). SAC induced the proliferation of both B-cell populations, while only CD5− B-cells were sensitive to signals delivered by anti-IgM. Following preactivation with SAC, IL-2 induced CD5− B-cells to secrete IgM, IgG, and IgA, while IgM was the predominant Ig isotype secreted by CD5+ B-cells (142). It has also been shown that stimulation of human fetal splenic CD5+ B-cells by anti-CD40 mAbs and IL-4 in the presence or absence of a T-cell clone resulted in the secretion of several Ig isotypes, but predominantly IgM (146). Moreover, a small subpopulation of peripheral blood CD5+ B-cells (between 0.5 and 2% of all peripheral blood CD5+ B-cells) appears to be class-switched to IgG or IgA (32). This finding indicates that isotype switching in CD5+ B-cells can occur *in vitro* and *in vivo*. Several studies in mice have provided evidence that CD5+ B-cells can undergo isotype switching (147, 148).

### Memory B-cell Subsets Are Not Homogeneous in Terms of Their *In Vitro* Differentiation Into Antibody-Secreting Plasma Cell

Human MBCs can be activated by CpG and cytokines without the need for BCR triggering or cognate interaction with T-cells (40, 149). *In vitro* studies have highlighted differences in the differentiation propensity between MBC subsets (16, 38, 149).

Bernasconi et al. (59) reported that switched and unswitched MBCs responded differently to CpG and bystander T-cell help (including CD40L–CD40 interaction). These authors showed that, in comparison to switched CD27+ MBCs, IgM+ CD27+ MBCs proliferated more efficiently in response to CpG ODN, but less efficiently in response to bystander T-cell help (59). They also showed that, in response to BCR and CD40L, unswitched MBCs (CD27+ IgD+) rapidly produced large amounts of predominantly IgM, while switched MBCs more rapidly initiated IgG and IgA synthesis (101). However, Marasco et al. recently reported contradictory results showing that, in response to CpG, switched MBCs proliferated more intensely than IgM MBCs and that stimulation with CD40L and anti-Ig did not induce any terminal differentiation or Ig secretion in either population (16). This study also showed that switched MBCs generated twice as many plasmablasts than IgM MBCs and that IgM MBCs only provided a minimal contribution to the pool of switched plasmablasts (16). However, overall, these studies agreed that IgM and switched MBCs show different functional capacities in response to CpG stimulation.

The IgM+ IgD+ CD27+ subset isolated from patients with hyper-IgM syndrome type I (characterized by CD40L gene mutations) has been shown to produce low levels of IgM in response to CD40L stimulation in the presence of IL-4 or IL-10 (150, 151). IgM+ IgD+ CD27+ B-cells differentiate into ASPCs—after CpG stimulation and secrete IgM and small amounts of IgG (38, 105, 110, 111); by contrast, naive B-cells do not differentiate into ASPCs (16, 111, 114). TLR9 stimulation has been shown to selectively expand IgM MBCs and promote differentiation of these cells into IgM-secreting PCs (106, 109). However, in the presence of IL-21, CD40L had no selective effect on MBCs and drove IgM as well as IgG secretion by these cells (109). Another study by Seifert et al. showed that IgM+ MBCs differentiated into PCs in response to anti-Ig stimulation (considered to be a T-cell-independent type of stimulation), as they adopted PC morphology and downregulated their expression of BTB and CNC homolog 2 (BACH2). However, following T-cell-independent stimulation (anti-Ig + CD40L), IgM+ MBCs show a preferential tendency to adopt a pre-GC B-cell phenotype, as indicated by upregulation of BCL6 transcripts (38). By contrast, human IgG+ MBCs primarily differentiated into PCs in response to either T-cell-dependent or T-cell-independent stimulation guided by rapid *PRDM1* (BLIMP1) induction and downregulation of BACH2 (38). These findings may be related to the ASPC differentiation-enhancing role of IgG BCR (152, 153). Furthermore, in contrast to human IgG+ MBCs and naive B-cells, co-culture of IgM+ MBCs with GM-CSF-activated neutrophils induces their differentiation into PCs (38).

Altogether, these observations indicate that the sensitivity of human B-cells to stimulation may reflect distinctive phenotypic, genetic, and functional B-cell subsets. A normal counterpart for malignant B-cells could be defined on the basis of shared phenotypic and/or genomic features. However, the phenotypic, transcriptomic, and genomic profile of CLL B-cells is different from that of any normal B-cell subsets that have been identified and studied, preventing the identification of a normal counterpart. Identification of the similar functional characteristics shared by normal B-cell subsets and CLL B-cells may contribute to the assignment of a normal cellular counterpart for CLL B-cells. In the next section of this review, we discuss the functional capacities of CLL B-cells and will try to define a normal counterpart based on functional perspectives.

## Normal Counterpart of CLL B-Cells: Current Approaches and Hypotheses

**Figure 2:**
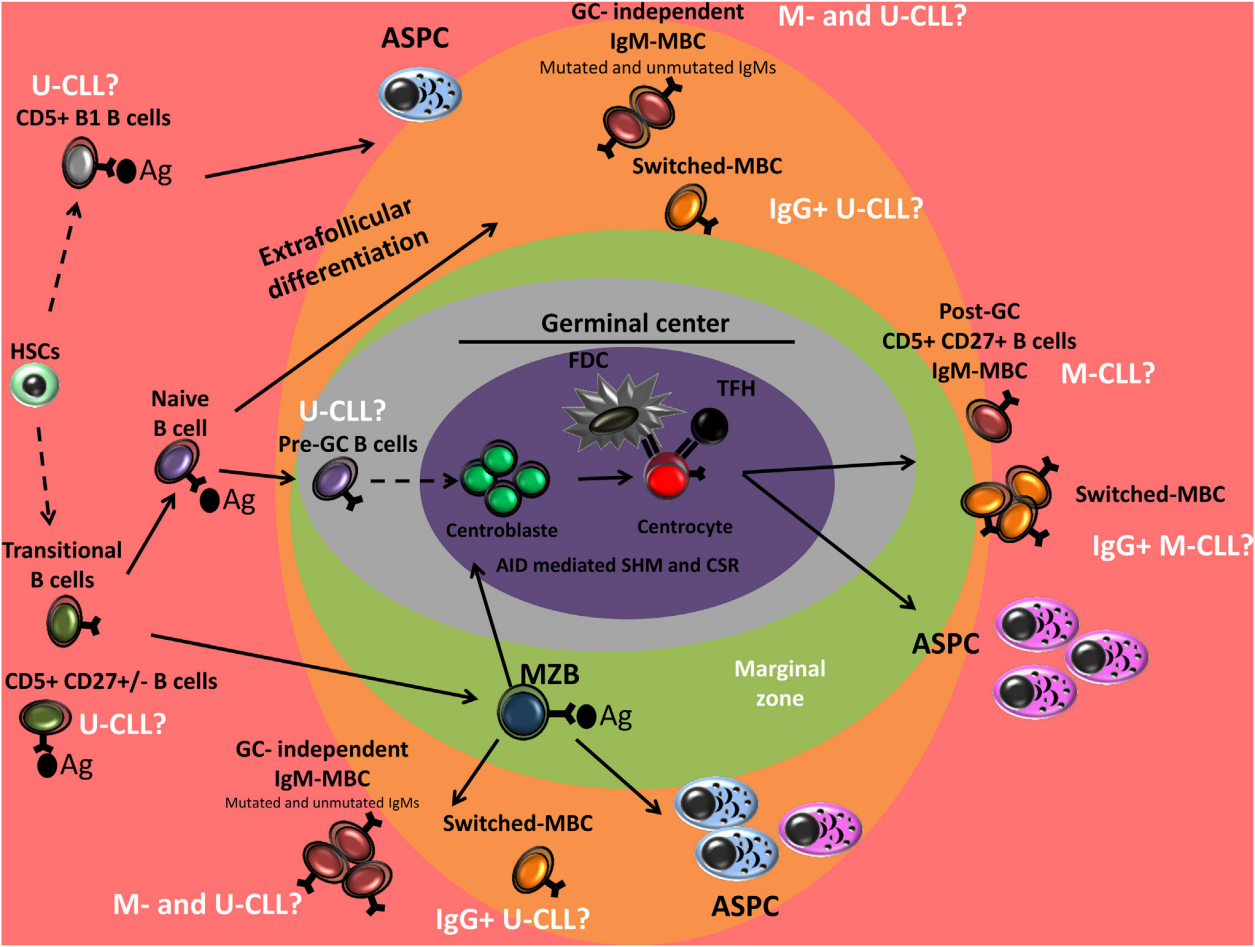
Peripheral B-cell development and normal B-cell counterpart of CLL. Genetic characterization of CD34+ hematopoietic stem cells (HSCs) and B-cell progenitors of CLL patients has shown the presence of the same mutations in the mature CLL B-cells, suggesting that CLL may originate in the early stages of hematopoiesis. Unmutated-CLL (U-CLL) and mutated-CLL (M-CLL) show similar gene expression profile. U-CLL present a gene expression and methylation profile similar to naive CD5+ B cells and M-CLL show profiles similar to post-germinal center (GC) CD5+ CD27+ MBCs. CLL B-cell could also derive from memory B-cells (MBCs) generated in GC-independent reaction. Unmutated CLL may derive from antigen-activated B-cells (conventional naive B-cells, CD5+ B-cells, or B1 B-cells). IgG-switched CLL B-cells are thought to have undergone class switch recombination (CSR) during the GC reaction, however, they are observed among both mutated- and U-CLL. From a functional point of view: (i) CLL B-cells were associated with (reversible) anergy, (ii) CLL B-cells can undergo somatic hypermutation (SHM) and CSR, (iii) B-cell receptor (BCR) stereotypy is observed in both mutated and unmutated CLL suggesting a role of antigen selection in pathogenesis of the disease, (iv) the Ag reactivity profile of CLL B-cells BCR overlap with that of natural antibodies and present reactivity against a wide range of pathogens, and (v) CLL B-cells are sensitive to TLR9 stimulation and show a high propensity for differentiation into PCs that predominantly secrete polyreactive IgM. These findings suggest that CLL B-cells could derive from B cell subsets that present these functional features including a fraction of human CD5+ B-cells, MZB-cells, human B1-like cells, and IgM MBCs. Ag, Antigen; MZB, marginal zone B cell; ASPCs, antibody-secreting plasma cells; CLL, chronic lymphocytic leukemia.

No consensus has yet been reached concerning the normal counterpart of CLL B-cells (Figure 2). It is also unclear whether CLL cells are derived from a single or multiple normal B-cell types (7). The earliest and current approaches in the search for the normal counterpart of CLL B-cells include morphologic assessment, immunophenotypic analysis, and molecular and epigenetic profiling.

Typical CLL B-cells exhibit a small cell body, with a normal-shaped nucleus with clumped chromatin surrounded by a thin ring of cytoplasm. These morphologic features are very similar to those of transitional/naive mature resting B-cells, as naive and MBC subsets differ in terms of their morphology (71, 101, 131, 154). Naive B-cells are small cells with scanty cytoplasm, while MBCs are predominantly larger cells with abundant cytoplasm on microscopy (101, 131) and flow cytometry (154). However, cases of atypical CLL cytology have been reported, corresponding to larger cells with more abundant cytoplasm, nuclear irregularities, and lymphoplasmacytoid or prolymphocyte features (155, 156).

Phenotypically, CLL B-cells almost always express IgM and IgD, CD5 and CD23 and can be CD38+ and CD10−. Prior to analysis of the IGHV gene mutational status and based on immunophenotypic analysis, CLL B-cells were thought to be derived from malignant transformation of follicular mantle-zone B-cells, which normally expresses CD5, CD23, coexpress membrane IgM and IgD, are negative for CD38 and use unmutated Ig V region genes (157). Demonstration of the increased expression of activation and costimulatory molecules (e.g., CD38, CD69, CD40, HLA-DR CD71, CD62L, and CD39) led to the hypothesis that CLL B-cells are derived from activated and Ag-experienced B lymphocytes (158). This concept is consistent with the uniform expression of CD27 on CLL B-cells, a marker of Ag-experienced MBCs (158, 159). By contrast, GC B-cells and MZB-cells were excluded; GC B-cells because they express CD10, CD38, lose IgD expression, frequently exhibit isotype switch and display somatic mutations in Ig genes (160) and MZB-cells because they are CD5-negative.

CD5 expression is an important feature of CLL B-cells and has been shown to inhibit BCR signaling and maintain tolerance in anergic B-cells after chronic (auto)antigenic stimulation (42) to limit autoantibody production. Furthermore, human CD5 has been shown to promote B-cell survival *via* autocrine IL-10 secretion by B-cells (161, 162) and is associated with RAG expression and receptor editing/revision outside GCs (163). CD5 expression could be transiently induced after activation of normal CD5− B-cells *in vitro* (42, 161, 163). Human CD19+ CD5+ B-cells include transitional T1 B-cell subset (28), a CD27+ transitional B-cell subset (46), a pre-naïve B-cell subset (30), a fraction of B1 B-cell subset (47), a mature naïve B-cells subset, and a CD27+ MBC subset (32). Recent analysis of human transitional B-cell subsets has led to the identification of a distinct population of CD27+ CD10low IgMlow CD5+ transitional B-cells (46). Several studies have identified a subset of CD5+ B-cells in the blood that co-express the memory-associated molecule CD27 (28, 32). These human CD5+ B-cells can undergo SHM, antigen selection, and possibly affinity maturation (164–168). CLL cells have an aberrant phenotype that includes IgM downregulation, reduced responsiveness to BCR ligands, reduced expression levels of CD21, above-baseline intracellular Ca^2+^, and activation of BCR pathway kinases, as well as negative feedback regulation, including SHP-1 activation similar to the phenotype of anergic autoreactive B-cells (169, 170).

Analysis of IGHV mutational status raised the possibility that CLL B-cells may be derived from two different cellular origins, unmutated CLL B-cells derived from pre-GC naive B-cells and mutated CLL B-cells derived from antigen-experienced, post-GC MBCs (7, 171). However, the earliest transcriptome analyses of CLL and normal B-cell subsets suggested that mutated and unmutated CLL B-cells display a homogeneous GEP that is largely independent of their VH mutation status and is more strongly related to MBCs than of cells derived from naive B-cells, cord blood CD5+ B-cells, or GC B-cells (centroblasts/centrocytes) (172). This finding was disputed recently by Seifert et al. who revealed that unmutated CLL clones were derived from mature, unmutated CD5+ CD27− B-cells and mutated CLL clones were derived from a distinct CD5+ CD27+ post-GC MBC subset (32). CD5+ CD27+ and CD5+ CD27− B-cells display a similar GEP, which may indicate that CD5+ CD27+ B-cells are derived from CD5+ CD27− B-cells that have undergone GC reactions (32). Normal CD5+ CD27+ B-cells and mutated IGHV CLL B-cells both harbor BCL6 mutations, a genetic trait of GC passage or AID expression and activity, supporting the hypothesis that IGHV-mutated CLL is derived from post-GC CD5+ MBCs. In the context of IGHV mutational status, pre- or post-GC B-cells have been proposed as the possible normal counterpart of CLL B-cells. Nevertheless, although SHM and MBCs are generally considered to be always generated in a GC-dependent manner, growing evidence in favor of the generation of AID-mediated SHM and MBCs in an GC-independent manner is emerging in humans (39, 62, 68, 69, 72, 73, 173–175) and in mice (54–56, 85, 89). In the light of these studies, the normal cellular counterpart of CLL B-cells should not though to only involve the pre- or post-GC B-cell subsets.

Comparison of miRNA expression profiles with those obtained for various normal B-cell subpopulations showed that the CLL miRNA expression signature most closely resembles that of normal antigen-experienced cells, including IgM memory and switched MBCs from peripheral blood and switched MBCs from tonsils (9).

Considerable research has been devoted to characterization of the CLL epigenome and has provided an overview of methylation changes in CLL B-cells compared to normal B-cells. Epigenetic programming of selective transcription factor binding sites was found to be correlated with the degree of B-cell maturation. A large-scale study of the epigenome of CLL and physiological B-cell population, based on similarities in methylation imprint, proposed naive B-cells as the putative normal counterpart of unmutated CLL and MBCs for mutated CLL (11). In another recent study conducted according to a similar experimental design, comparing the sequence and chromatin features of genomic regions that are programmed in normal B-cell maturation versus CLL B-cells, Oakes et al. proposed a less categorical view (not restricted to discrete maturation stages) and proposed the hypothesis that the heterogeneity of the disease is related to a continuum of maturation states of the normal counterpart, corresponding to the normal developmental stages of B-cells (between early unswitched MBCs and switched MBCs) (12).

A major limitation to studies investigating the normal counterpart of CLL B-cells is the clear distinction between characteristics that are CLL-specific and those that are derived from the cell-of-origin. As malignant lymphocytes are considered to maintain their key programmed features of the stage of differentiation of their normal cellular counterpart, a functional approach could be a good way to eliminate disease-specific features and access a new state that is independent of disease characteristics.

## What Can the Functional Features of CLL B-Cells Tell Us about Their Normal Counterpart?

### Anergy

One functional feature attributed to CLL B-cells is (reversible) anergy (169). The molecular signature of anergy has been detected in both unmutated and mutated CLL B-cells (176). Anergic B-cells are also characterized by constitutive activation of MEK, ERK, and nuclear factor of activated T cells (NF-AT) in the absence of Akt phosphorylation and low membrane BCR expression, features observed in CLL B-cells lacking an induced BCR signaling capacity (176). The lower levels of sIgM expression by CLL B-cells (essentially the mutated subset) are associated with failure to respond to *in vitro* sIgM engagement (177). The encounter of CLL B-cells with stimulating agents in the tissue microenvironment reverses anergy and may initiate proliferation (169). *In vitro* culture or stimulation of CLL B-cells {for example, by TLR-ligand or cytokines [IL-4 (178)], or encounter with T cells} may reverse the anergic status and upregulate cellular expression of surface IgM (177, 178). Anergy is a mechanism of immunological tolerance that censors autoreactive B-cells and reminiscent of B-cells that have undergone receptor desensitization following chronic antigenic stimulation. These data suggest that CLL cells may derive from poly-/autoreactive B-cells.

### Isotype Switching in CLL B-Cells

Despite the SHM-based subcategorization of CLL cases and the expression of surface IgM and IgD in the majority of cases, some clones exhibit ongoing IGHV diversification and CSR *in vivo* [CSR (179–184), SHM (185–188)] and some cases present an antigen-driven pattern (189). Freshly isolated sIgM+ sIgG− sIgA− CLL B-cells express IgG and IgA transcripts that have identical VDJ segments (179, 181). Earlier studies have described isotype switching in CLL B-cells following *in vitro* stimulation (190–192). SAC and conditioned T-cell culture supernatant were shown to induce the production of IgG by the cells of CLL patients (190, 191). Culturing leukemic B-cells in the CD40 system in the presence of IL-10, but not IL-4 or transforming growth factor-β (192), induced CLL B-cells to switch to IgG and IgA (181). Isotype switching of CLL B-cells following differentiation into PCs has been observed in several *in vitro* studies, but CLL B-cells predominantly differentiated into IgM-secreting PCs (14, 18–21, 193–196). Cases of CLL in which the major clone expresses Ig isotypes other than IgM and IgD, for example, IgG or IgA (197, 198) are relatively rare (5%) (199). These Ig-switch CLL are observed in both mutated and unmutated CLL, challenging the scenario of a post-GC origin (200). Studies in CD5+ IgG+ CLL B-cells found a skewed Ig gene repertoire with overuse of the IGHV4-34 and IGHV4-39 genes and a higher SHM load (197, 200, 201). Of interest, a small subpopulation of normal human CD5+ B-cells (between 0.5 and 2% of all CD5+ PB B-cells) that use these VH genes appears to be class-switched to IgG or IgA (32). These data indicate that CLL B-cells may be derived from a B-cell subset that can undergo SHM and CSR.

### IgHV Mutational Status and Stereotypy

Immunogenetic analysis revealed that both mutated and unmutated CLL present a highly restricted and biased repertoire of Ig genes indicating a role of antigen selection in pathogenesis of the disease (202, 203). This phenomenon is observed in 30% of CLL patients (203) and is known as BCR stereotypy, based on the structural similarities of their complementarity-determining regions. More than 200 different CLL stereotyped subsets have been identified to date, with 19 major subsets accounting for 40% of all stereotyped cases and 10% of all CLLs (203). Stereotypy seems to be a random process in healthy individuals, however, a biased toward a restricted number of Ig genes is observed in CLL, including IGHV1-69, IGHV3-7, IGHV3-21, and IGHV4-34 (171, 202).

Immunoglobulin heavy-chain variable gene family usage differs between human B-cell subsets and can be modified by age (204), as the relative use of IGHV1 and IGHV3 genes seems to be a marker that can be used to distinguish between a number of different B-cell types (204). IGHV1 family gene usage increases and IGHV3 family gene usage decreases between naive and switched MBC repertoires. MBC subsets have distinct repertoire characteristics with an increase in the IGHV3 family at the expense of the IGHV1 family in IgM memory cells (205). Conversely, class-switched B-cells are characterized by increased IGHV1 and decreased IGHV3 (205). Transitional B-cells showed an increase in the IGHV3 family at the expense of IGHV1 compared to naive B-cells (26). The distinctive pattern of IGHV gene use by B-cell subpopulations may be indicative of different selection pressures in an immune response. However, the existence of BCR stereotypy in the normal repertoire was evidenced by the identification of stereotypic IGHV1-69/IGHJ6 rearrangements (that constitute 13% of all CLL and 25–30% of unmutated-CLL) in circulating naive B-cells of healthy elderly individuals (206).

### Multiple Clones and Multiple Mutational Statuses in CLL

Next-generation sequencing has allowed more detailed analysis of IGHV status and the presence of multiple IGHV rearrangements has been demonstrated in the same patient in up to 5–24% of all cases of CLL (187, 188, 207–209), possibly as a result of the lack of allelic exclusion and the presence of two productive rearrangements or it may be correspond to the presence of multiple leukemic subclones. Furthermore, in line with these observations, the identification of multiple clones with different mutational status in CLL (187) may suggest that SHM is an ongoing event in CLL that can occur in an Ag-independent or -dependent manner. The monoclonal B-cell lymphocytosis precursor state, which precedes the clinically relevant leukemic phase in virtually all CLL patients, therefore often involves multiple B-cell clones that sometimes show ongoing VH gene mutations (209).

### Antibody Reactivity

The idea of a common antigen driving the disease is supported by the sharing of stereotyped BCR. Consistent with this idea, studies of structural data and modeling of light and heavy chain variable region pairs from over 300 CLL patients revealed a restricted series of predicted antigen-binding sites, suggesting that a restricted number of antigenic structures may be implicated in the pathogenesis of CLL (210).

The Ag reactivity profile of CLL B-cells BCR appears to overlap with that of natural Abs produced in the absence of exogenous Ag stimulation and that play a crucial role in immediate host defense against a wide range of pathogens (211–213). Since the 1980s, it is known that CLL B-cells produce polyreactive and autoreactive Abs, capable of binding human Ig, or single- or double-stranded DNA (214, 215). Recent studies of the Abs expressed by CLL B-cells from both mutational subgroups, with or without stereotyped BCRs, have identified common antibody reactivity to a number of self-Ags, predominantly cytoskeletal proteins (non-muscle myosin heavy chain IIA, vimentin, filamin B, and colifin-1), cardiolipin, and oxidized low-density lipoprotein (212, 216). Recently, defined epitopes within the BCR third complementarity-determining region of the heavy chain have been reported as targets of BCR self-recognition in CLL, representing an alternative form of self-antigen (170, 217). In addition to self-Ags, a number of CLL Abs have also been demonstrated to exhibit specificity for bacterial antigen capsules and toxins (including *S. pneumoniae* polysaccharides, *S. aureus* protein A superantigen) as well as viral coats and fungi (211, 212, 218–220). In a recent study, Hatzi et al. (221) found that unmutated CLL BCR were much more broadly bacterial reactive than mutated CLL BCR.

Although polyreactivity has been previously described in about 80% of unmutated CLL cases and only 15% of mutated CLL (211), Herve et al., in an *in vitro* study, reverted mutated CLL B-cell Abs to their original germline sequences (non-mutated) and showed that they encode poly- and autoreactivity Abs (211). These data have led to the hypothesis that both mutated and unmutated CLL may arise from a common population of B-cells, which produce low-avidity, polyreactive, “natural antibodies” that may participate in a maintenance function by eliminating apoptotic cells, and contribute to the initial stages of the immune response to foreign pathogens (211, 212). Among the various human B-cell subsets, these types of Abs may be secreted by naive mature B-cells, MZBs, and IgM MBCs (60, 61) (when resulting from extrafollicular differentiation, see above), but also human B1-like cells (49) and immature/transitional B-cells.

### AID Expression

Activation-induced cytidine deaminase drive antibody affinity maturation by incorporating point mutations over the rearranged variable region of the antibody in antigen-activated B-cells *via* the mechanism of SHM and initiates the DNA breaks that trigger CSR (174). Both SHM and CSR are by no means restricted to GC sites. Extrafollicular differentiation in response to TD and TI Ag can generate IgM MBCs and ASPC with low-frequency SHM (39, 60, 61, 69–71). Furthermore, AID can be expressed by transitional B-cells and has been shown to be essential for central B-cell tolerance and to remove autoreactive clones (137, 138). AID-deficient and AID-mutated patients present an abnormal peripheral B-cell tolerance checkpoint and a high frequency of autoreactive mature naive B-cells (222). In humans, IgM MBCs and MZB-cells also undergo SHM in the absence of immunization *via* incompletely understood mechanism that becomes active at a very early developmental phase (39, 73, 173, 175). Consistent with the moderate mutational load in their expressed IGHV genes, MZB-cells contain molecular footprints of past proliferation in an extrafollicular environment (39, 71, 73). Furthermore, *in vitro* transitional B-cells can differentiate into ASPC, can express AID, and can acquire somatic mutations (137).

The presence of AID transcripts and protein has been described in both mutated and unmutated CLL (182, 223). AID protein has been detected in proliferating CLL B-cells residing in lymph nodes or in response to *in vitro* stimulation of peripheral blood CLL B-cells (223). *In vitro* stimulation of CLL B-cells by CD40L in the presence of IL-4 induced AID expression and showed AID-dependent diversification of their IgV genes and triggered CSR (182, 223). AID protein and its functional consequences (CSR and SHM) have also been observed in CLL xenografts in mice (17).

Aberrant AID activity can lead to mutations, deletions, or translocations outside of the Ig locus. AID promiscuously targets a subset of transcriptionally active genes, including the proto-oncogenes BCL6. The BCL6 proto-oncogene has often been found to be mutated at proximal promoter sequences in GC-derived B-cell lymphoma. The presence of BCL6 mutations in CLL B-cells has been used as an argument for GC passage. In fact, BCL6 mutations may be a marker of AID activity, but not necessarily GC passage. Furthermore, CLL B-cells lack the chromosomal translocations observed in most GC malignancies [e.g., t(14;18) in follicular lymphoma] and that are linked to AID activity (174). CLL B-cells may also arise from early GC B-cells or post-GC B-cells, in which Ig class switching and SHM are no longer active.

### CLL B-Cells and Terminal Differentiation Into Antibody-Secreting Plasma Cell

**Figure 3:**
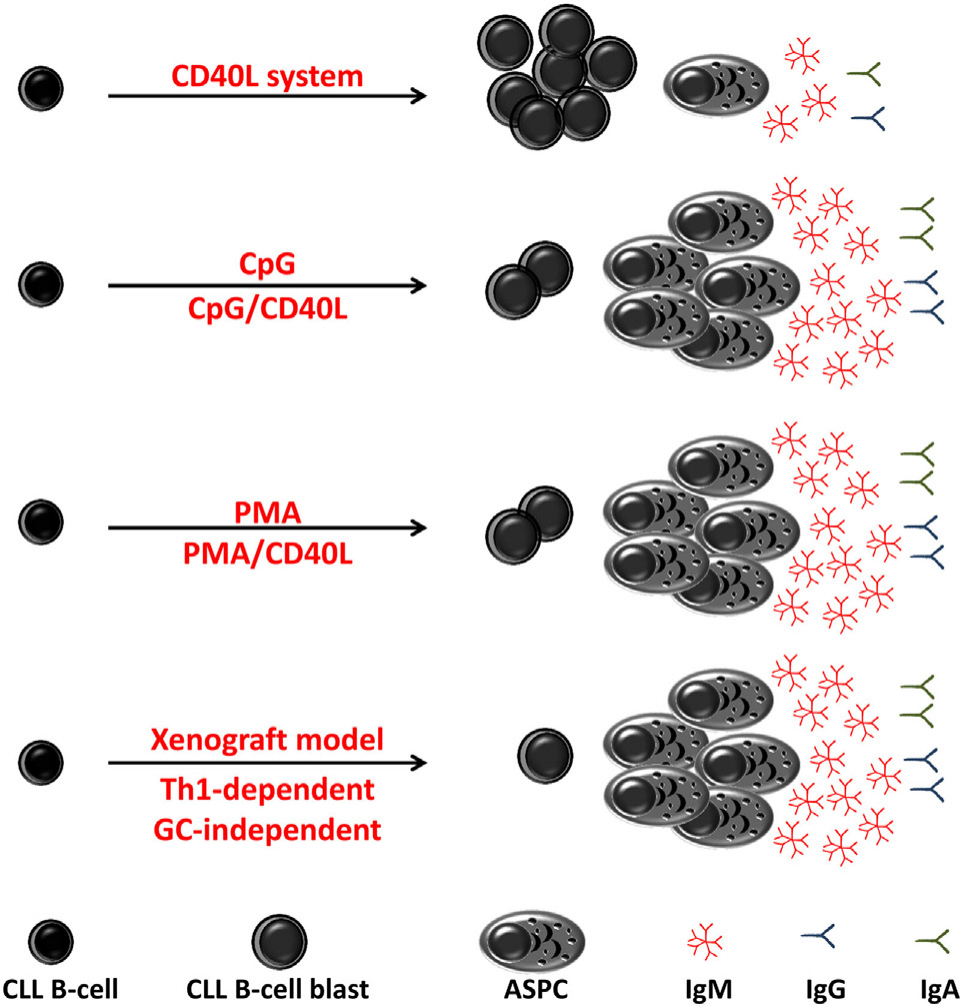
Differentiation of chronic lymphocytic leukemia (CLL) B-cell *in vitro* and *in vivo*. *In vitro* and *in vivo* (xenograft model) CLL B-cells differentiate into antibody-secreting plasma cells (ASPCs) with different efficiency. In response to stimulation through CD40 ligation (CD40L), CLL B-cells poorly differentiated into antibody-secreting plasma cell (ASPC) and showed an activated phenotype and morphology and low levels of Ig secretion. In response to CpG or to phorbol myristate acetate (PMA), CLL B-cells show a high propensity for differentiation into ASPC that predominantly secrete IgM. In xenograft model, differentiation of CLL B-cells has been shown to be induced by T helper 1-polarized T-bet+ T-cells and not classical T follicular helper cells, i.e., in the absence of germinal center.

The functional features of terminally differentiated B-cells (e.g., ASPC) are in some way programmed into and inherited from the first cell (Figure 3). Human B-cell subsets display an intrinsically altered propensity to proliferate and differentiate into ASPC after exposure to the same stimuli. There is accumulating evidence in support of the stimulation requirements and differentiation potential of normal B-cell subsets. Defining and comparing these capacities, requirements, and propensities between CLL B-cells and normal B-cell subsets may help to guide assignment of the normal counterpart of CLL B-cells. For example, some very early data showed that crosslinking of BCR on the cell surface using anti-μ or SAC induces terminal differentiation of CLL B-cells, but not that of normal B-cells from peripheral blood (191, 224). However, when stimulated with the polyclonal activator, pokeweed mitogen (PWM), normal B-cells differentiate into IgM- and IgG-secreting cells and CLL B-cells essentially differentiate into IgM-secreting PCs (193). These studies highlight intrinsic differences in the requirements for terminal differentiation between CLL B-cells and normal B-cells.

### Earliest Work

*In vitro* activation and differentiation of leukemic CD5+ B-cells have now been investigated for about 50 years and have been used as a model for normal B-cell development and to study the role of different growth factors in the pathogenesis of B-cell malignancies (22, 225–228). In the 60s, the inability of CLL cells to switch to a hyperbasophilic cell morphology after mitogen stimulation (such as PHA, considered, at the time, to be a ubiquitous antigen, a growth factor, and a stimulator of gamma globulin secretion) was interpreted as evidence of their “immunoincompetence” and responsible for their accumulation (225, 229). Furthermore, the “immunoincompetence” of CLL cells was underlined by the low production of Abs in CLL patients vaccinated against typhoid, influenza, and diphtheria (225). In 1973, an electron microscopy study showed transformation of CLL cells into a plasmacytoid morphology after stimulation with PWM (230). In a study published in 1974, the authors demonstrated that the Ig expressed at the surface of isolated leukemic cells from a CLL patient was idiotypically identical to serum monoclonal Ig (an IgM) from the same patient. The authors hypothesized that serum Ig is produced by PCs differentiated from leukemic cells (23). Concomitant PC malignancies and CLL have been reported in several studies (231, 232). Several studies showed that differentiation of CLL cells into ASPCs can occur spontaneously *in vivo* (23, 24). In some cases, the authors showed a clonal relationship between the two malignant cells resulting from differentiation of CLL B-cells into PCs (23, 24, 233–235).

Chronic lymphocytic leukemia cells were thereafter used as model to study B-cell differentiation (226–228). Thus, in the 1980s and 1990s, many *in vitro* studies were devoted to the differentiation of CLL cells into ASPCs in response to stimulation by different mitogens (22, 194, 236–242). However, remarkable results were obtained with a phorbol diester, phorbol myristate acetate (PMA). This molecule has been shown to induce differentiation and activation of CLL cells into ASPCs (22, 194, 236–238, 241) [reviewed in Ref. (242)], suggesting a critical role for the protein kinase C (PKC) pathway in the activation and differentiation of CLL B-cells (see below).

### CLL B-Cells Resemble Subpopulations of Normal B-Cells That Are Responsive to Phorbol Ester

Phorbol myristate acetate [also known as 12-*O*-tetradecanoylphorbol 13-acetate (TPA)] activates the PKC pathway by mimicking diacylglycerol (DAG), a natural ligand and activator of PKCs (242). PMA mimics DAG, the direct physiologic activator of PKC. PKC pathway is importantly implicated in B-cell development and function (242). PKC is expressed in CLL and has been shown to be involved in the pathogenesis of CLL (243). PMA induces differentiation of CLL B-cells in a T-cell-independent manner (22, 194, 196). PMA show a specific effect on CLL B-cells, with no effect on other B-cell malignancies, and this effect is enhanced in the presence of IL-4 and T-cell-derived cytokines (21, 22, 195, 196, 239–242, 244, 245). Following PMA activation, CLL B-cells undergo “plasmacytoid” transformation, with an increase in cell size, cellular RNA content, cytoplasmic Ig and IgM secretion, enhanced allostimulatory activity, and cell cycle entry (242, 244–246). These results using PMA were reproduced by recent studies (21, 196). Ghamlouch et al. (196) used PMA in combination with cytokines IL-2, IL-4, IL-10, and IL-12 in the presence or absence of CD40L to induce CLL B-cell differentiation into ASPCs. Generated ASPCs presented (i) high levels of cytoplasmic IgM expression, (ii) induction of the unfolded protein response (UPR), reflected by upregulation of UPR targets and the protein folding machinery of the endoplasmic reticulum (ER): GRP78/Bip and GRP94 and calnexin, and (iii) enhanced secretion of IgM. Furthermore, PMA-induced CLL B-cell differentiation into ASPC occurs in the absence or in the presence of very low levels of DNA synthesis and cell proliferation (21, 247). Ghamlouch et al. (21) showed that cell cycle entry during CLL B-cell differentiation into ASPC in PMA- or TLR9-based systems is very low (<5% in S-phase) compared to that observed for differentiating cells in a normal naive and memory human B-cell differentiation system (between 15 and 35% in S-phase) (99, 106, 108, 115).

Phorbol myristate acetate alone or in combination with calcium ionophore and cytokines has been shown to induce proliferation and activation, but little or no terminal differentiation of human normal B-cells from peripheral blood and tonsil (248–251). The early response gene regulatory pathways have been shown to differ between normal and CLL B-cells in response to PMA activation (236). However, some studies have indicated that only a small fraction of normal B-cells undergo terminal differentiation into ASPC in response to PMA (236, 248, 252, 253). To our knowledge, no study has investigated the type of normal B-cells that differentiate in response to PMA. These studies suggested that CLL B-cells may resemble these minor subpopulations of normal B-cells that are activated and differentiate *in vitro* directly *via* the PKC pathway (236, 252, 253). Furthermore, after PMA treatment, a fraction of human B-cells has been shown to express CD5 molecules on their cell surface (254). In contrast with normal B-cells (from peripheral blood and tonsil), PMA is able to activate CLL B-cells, as evidenced by the expression of cell surface activation markers, and to induce their terminal differentiation into IgM-secreting PCs together with low DNA synthesis and proliferation (247, 249).

### TLR-Induced Antibody-Secreting Plasma Cell Differentiation of CLL B-Cells

Researchers have recently studied the role of TLR stimulation on CLL B-cell activation and differentiation. Several studies have reported that TLR7 and TLR9 stimulation (mainly focused onto TLR9) shape an immunogenic phenotype in CLL B-cells (255). CLL B-cells express high levels of TLR9 (255, 256). Similar to PMA, treating CLL B-cells with the TLR9 agonist CpG ODNs induces significant morphologic changes and phenotypic activation, as shown by increased cell surface expression of CD54, CD86, and HLA-DR and intense proliferation of allogeneic T-cells in mixed lymphocyte reaction (256). The proliferative response to CpG stimulation alone has been shown to be reduced compared to normal peripheral B-cells. The combination of CpG with cytokines such as IL-2 or IL-15 has been shown to promote *in vitro* proliferation of CLL B-cells and prevent apoptosis (257, 258). Activation and proliferation following stimulation with TLR9 ligand differs between B-CLL cells and normal B-cells in terms of IL-6 production, CD40 expression, and regulation of early cell cycle progression (e.g., regulation of cyclin D3 and p27 expression) (257, 258). TLR9 activation induced CD5 expression on the cell surface of normal B-cells and upregulated the Syk family tyrosine kinase ZAP-70 predominantly in the IgM+ MBC subset (110). ZAP-70 has been shown to be expressed in a subpopulation of normal tonsillar and splenic B-cells that express CD5, CD27, and CD38 (259).

Toll-like receptor 9 signaling has been shown to induce differentiation of CLL B-cells and antibody secretion when costimulated with cytokines (14, 18–20). Gutierrez et al. showed that CpG, in combination with cytokines IL-2 and IL-15, induces differentiation of CLL B-cells into IgM-secreting PCs (18). Duckworth et al. showed CpG and IL-21 to be useful differentiation-promoting agents in CLL B-cells (20). In a two-step, 7-day culture system, Ghamlouch et al. showed that CLL B-cells can differentiate into CD20+ plasmablasts/ASPCs when stimulated with a combination of CpG, CD40 ligand, and cytokines (14). In a 28-day culture system, Hoogeboom et al. observed that CLL B-cells can differentiate into IgM-secreting PCs when stimulated with CpG alone or in combination with CD40L (19). Similar to PMA-induced differentiation (21, 196), differentiation *via* TLR9 signaling induced CLL B-cells to express the PC transcriptional program (e.g., STAT3, IRF4, XBP1s, and BLIMP1) and downregulated expression of B-cell transcriptional programs, including c-MYC, PAX5, BCL6, IRF8, and BACH2 (14, 20, 21).

Varying degrees of differentiation were observed in different patients, and this effect was associated with functional anergy of leukemic cells and epigenetic aberrations (transcriptional repression of PRDM1 gene) (19, 20). Researchers have also observed reversal of CLL cell anergy by appropriate *in vitro* culture associated with the capacity to induce *PRDM1* (coding BLIMP1) expression in response to appropriate stimulation. The heterogeneity of responses could be due to the origin and preparation of CLL B-cells. Heterogeneity of responses has been observed in studies not using isolated CLL B-cells [whole peripheral blood mononuclear cells (PBMC)] or not using freshly isolated CLL B-cells (cryopreserved CLL cells). It has been shown that the presence of non-B-cells in culture influences the differentiation responses of memory and naive B-cells (16). The frequencies of viable CLL B-cells (when using cryopreserved CLL cells) or the activation of myeloid cells and T-cells by the stimulating agents (when using PBMCs) can affect the differentiation and Ig secretion of CLL B-cells. However, this heterogeneity could also be linked to the CLL B-cell of origins (the B-cell type). Nevertheless, donor-to-donor variability is also observed for normal B-cell differentiation responses.

High TLR9 expression is a feature of CLL B-cells. TLR9 is also highly expressed by transitional B-cells and MBCs (particularly IgM MBCs) (105). In a TLR9 pathway-based differentiation system, CLL B-cells showed efficient differentiation into IgM-secreting PCs and were shown to undergo isotype switching similar to that observed for MBCs (including IgM MBCs), transitional B-cells but not naive peripheral blood B-cells. Furthermore, TLR9 activation induced IgM+ CD27+ B-cells to express CD5, CD23, CD25, HSP90, and ZAP70 similar to CLL B-cells, features not observed in response to CD40L (110). Overall, these observations could reflect differences in functional capacity (signal transduction pathways, activation requirement, and propensity for terminal differentiation) between major B-cell subpopulations and CLL B-cells and suggest that only a minor subpopulation of normal B-cells are functionally similar to CLL B-cells and could represent a potential normal counterpart of these cells.

### Role of T-Cells in Activation and Differentiation of CLL B-Cells

There is evidence that T helper 1 (Th1) cells can support CLL activation and proliferation (17, 260–263). In CLL, lymph nodes and bone marrow contain large numbers of CD4+ T lymphocytes (263), many of which express the costimulatory molecule CD154 (CD40L) (263). CD40-activated CLL B-cells exhibit an activated B-cell phenotype and morphology (192, 196, 264, 265), with upregulated IRF4 expression, decreased expression of CD20 and CD184, and enhanced expression of CD18, CD40, CD54, CD80, CD86, and HLA-DR, and secrete moderate levels of IgM (196, 265, 266). CD40L treatment or co-culture of CLL B-cells with CD4+ T lymphocytes has been demonstrated to increase CLL B-cell survival, at least partly mediated *via* IL-4 signaling (267, 268).

Soluble CD40L/anti-CD40 and T-cell-derived cytokines were used to mimic T-cell help *in vitro* (CD40 system) and study B-cell subset differentiation in response to T-cell help. In this system, CLL B-cells poorly differentiated into ASPC and showed an activated phenotype and morphology and low levels of Ig secretion (196, 265, 266). However, the combination of TLR9 ligand or PMA with CD40L potentiated the ASPC differentiation of CLL B-cells (14, 19, 21, 196). Switched and IgM MBCs showed efficient differentiation into IgM-, IgG-, and IgA-secreting cells (38, 59, 99, 102, 104–107, 123, 125, 135). Nevertheless, peripheral blood transitional B-cells [including CD5+ CD27+ CD23+ B-cell subsets and CD5+ CD27− pre-naive B-cells (30)] and mature follicular naive B-cells are low responders in terms of CD40L-induced differentiation similar to CLL B-cells. These data indicate that CLL B-cells do not share the same activation and differentiation requirements of “classical” MBCs (99, 104, 123). By contrast, CLL B-cells behave like transitional or mature naive B-cells in a CD40 system, in which a small proportion of these B-cell subsets are induced to differentiate into IgM-secreting PCs (99, 104, 123).

Xenograft studies have recently highlighted the role of T-cell in activation, proliferation, and differentiation of CLL B-cells (17, 261). In a xenograft mouse model, Os et al. (261) showed that CLL B-cells are induced to proliferate by autologous Th cells and this activation was CD40L dependent. Conversely, autologous Th cells can be efficiently activated by CLL B-cells in a endogenous CLL antigen-specific manner, as CLL B-cells act as efficient antigen-presenting cells of endogenous Ags both *in vitro* and *in vivo* (261). Exogenous antigen-driven Th cell–CLL cell interaction has been shown to drive CLL B-cell activation and differentiation into IgM-secreting PCs that did not downregulate HLA and costimulatory molecules or express the PC marker CD138. Interestingly, these features were observed in the *in vitro* TLR9 and PMA differentiation system of CLL B-cells (in the presence and absence of CD40L) (14, 18, 20, 21, 196, 265).

Chronic lymphocytic leukemia-specific Th cells have been shown to be IFN-γ secreting Th1-like cells that express the IFN-γ-associated transcription factor T-bet (261, 269). The T-bet transcription factor is also expressed by CLL cells (261, 270). Recent studies have shown that T-bet can directly bind to Bcl6 and repress Bcl6 target gene expression in T-cells (87, 88). Furthermore, a recent study in mice showed that Th1 cells are capable of providing sufficient help to B-cell in a GC-independent response to generate a protective antibody response to influenza infection (86). Very recently, in a xenograft mouse model, Patten et al. showed that human malignant B-cells from CLL patients differentiated into ASPCs in the presence of patient-derived T-cells (17). Terminal differentiation was induced in mutated and unmutated CLL B-cell clones by Th1-polarized T-bet+ T-cells, but not classical TFH cells. In this study, CLL B-cell differentiation was associated with upregulation of IRF4 and BLIMP1 with no measurable levels of BCL6, features also observed in the *in vitro* TLR9, PMA, and CD40 differentiation systems of CLL B-cells (14, 18, 20, 21, 196). Furthermore, differentiation was associated with isotype class switching and development of new IGHV-D-J mutations that involve AID in both unmutated and mutated CLL B-cells (17). In these models, data indicate that differentiation of CLL B-cells occurs in an extrafollicular/GC-independent manner, in which SHM and CSR can occur (34, 58, 84–86) and T-cells can play a cytokine-mediated role. These findings suggest that mutated and unmutated CLL B-cells are both derived from a B-cell subset that is able to differentiate in an extrafollicular manner essentially into IgM-antibody secreting cells.

## CLL B-Cell Normal Counterpart: Implications of Monoclonal B-Cell Lymphocytosis (MBL) and HSC

Monoclonal B-cell lymphocytosis (MBL) is defined as the presence of a circulating monoclonal B-cell population with B-cell count below than 5 × 10^9^/L and no other signs of a lymphoproliferative disorder (271). The majority of cases of MBL present a CLL immunophenotype and may present the chromosomal abnormalities observed in CLL. Landgren et al. showed that 44/45 patients with CLL had a precursor MBL state that had been identified between 6 months and 7 years prior to the diagnosis of CLL (272). The risk of progression of high-count MBL (clonal B-cell count between 0.5 and 5 × 10^9^/L) to CLL or small lymphocytic leukemia requiring therapy is between 1 and 2% per year (271). Of note, MBL is more frequently composed of two or more coexisting B-cell clones (20–70% of total cases) than CLL (209, 273). Progression from MBL to CLL could be a stepwise process, in which a single dominant clone progressively expands following positive selection by an antigen or somatic mutations. Recent evidence suggests that, in this model, the first oncogenic event could be traced back at least to the progenitor with no IGHV gene rearrangements or HSC (3, 274). In line with this hypothesis, Damm et al. demonstrated that some CLL driver mutations (e.g., SF3B1 or NOTCH1) are shared between the mature CLL clone and HSCs and hematopoietic progenitor populations, demonstrating that these mutations occur in a progenitor able to undergo lymphoid and myeloid differentiation (3, 275). Identification of multiple clones with different IGHV rearrangements in CLL also suggests that leukemia-initiating events might occur prior to IGHV rearrangement in an un-rearranged early B-cell progenitor (187, 188, 208). Moreover, in a xenograft model, HSPCs from CLL patients presented increased susceptibility to generate expansions of (oligo-mono) clonal B-cells carrying V(D)J gene rearrangements, which were always unrelated to those of the original CLL cells, indicating that HSPCs from CLL may exhibit abnormal B-cell differentiation and suggesting that self-renewing HSPC may constitute the CLL cell-of-origin (274). Mature B-cells resulting from this hematopoiesis could then be selected *via* the interaction with Ags (autoantigen, classical Ags, and/or superantigens), resulting in the expansion of mono- and/or oligoclonal B-cell populations. With age (276–278), additional genomic abnormalities would occur (disrupting the control of cell growth and survival), autoantigen availability would increase due to increased cell death and clearance deficiency, and susceptibility to infections would increase due to age-associated immune dysfunction. These changes could participate in the development of clonal MBL and/or CLL disorders. How can a B-cell normal counterpart of CLL B-cells be defined in such a complex model of pathogenesis combined with the complexity of human B-cell differentiation into the various mature B-cell subsets?

## Conclusion

In all studies investigating differentiation of CLL B-cells into ASPC, CLL B-cells show a high propensity for differentiation into PCs that predominantly secrete IgM (Figure 3). These data indicate that CLL B-cells originate from a B-cell compartment that rapidly differentiates into predominantly IgM-producing ASPCs with little isotype switching. These features have been observed for naive mature follicular B-cells (when differentiate in an extrafollicular manner), transitional B-cells (including a fraction of human CD27+/– CD5+ B-cells), MZB-cells, human B1-like cells, and IgM MBCs. As mentioned above, all B-cell subsets do not present the same responses to TLR9 ligation in terms of PC differentiation. CLL B-cell differentiation is sensitive to TLR9 stimulation. Among the various B-cell subsets, naive mature B-cells are unresponsive to the TLR9 differentiation pathway, while transitional B-cells, IgM MBCs, and MZB-cells respond to TLR9 stimulation and differentiate into ASPC predominantly secreting IgM but also some IgG and IgA, similar to CLL B-cells. CLL B-cells produce low-avidity, polyreactive, “natural” Abs (211, 212). The secretion of these kinds of Abs is usually attributed to MZB-cells (60, 61), but also human B1-like cells and immature/transitional B-cells.

*In vivo* (17) differentiation of CLL B-cells has been shown to be induced by Th1-polarized T-bet+ T-cells and not classical TFH cells, i.e., in the absence of GC. These data suggest that CLL B-cells originate from a B-cell subset that differentiates in a GC-independent manner (15). Interestingly, this differentiation pathway is that described for IgM MBCs, MZB-cells, and B1 cells (54–56, 62), but could also be that of transitional B-cells, as suggested by *in vitro* and murine studies (46, 111, 121, 124, 140, 141).

In depth, characterization of human transitional B-cells has led to the identification of distinct populations, anergic type 3 B-cells, and CD27+ transitional B-cells (46). Anergic transitional B-cells were CD10low IgMlow CD5+, unresponsive to IgM ligation and did not differentiate into ASPC after TLR9 engagement. CD27+ transitional B-cells were shown to exhibit an activated phenotype and express CD5, CD23, and TLR9 (46). These CD27+ transitional B-cells produce IL-10 and show a high capacity to differentiate into IgM-secreting PCs and MBCs upon TLR9 stimulation and can regulate T-cell proliferation (46). These features (including IL-10 secretion) are also described for CLL B-cells (14, 162, 261, 279). Moreover, human transitional B-cells have been shown to express T-bet, AID (137, 140), and high levels of lymphoid enhancer-binding factor 1 (31) similar to CLL B-cells. As transitional B-cells were excluded from molecular studies addressing the CLL B-cell normal counterpart (11–13, 32), it would be interesting to compare the gene expression, miRNA, and epigenetic profile of transitional B-cell subsets (at least the CD5+ subset) with that of CLL B-cells.

Over recent years, several studies have presented evidence suggesting that the earliest genetic and epigenetic events in the pathogenesis of CLL might occur in HSCs. However, it is now widely accepted that B-cell development progresses linearly through HSC → Pro-B → pre-B → immature → transitional → naive mature B-cell subsets, including follicular and MZBs and after Ag encounter → the various PC and MBC subsets. Despite the heterogeneous landscape of somatic mutations in CLL, CLL B-cells have a similar phenotype and GEP. By taking this into account, in a stepwise leukemogenesis model from the HSC or B-cell progenitor to the transformed CLL B-cell, leukemic transformation more likely occurs at a B-cell stage of development with homogeneous phenotype and GEP. The stage most likely to present these parameters is a B-cell stage located before the bifurcation into the various mature B-cell populations (a subset of transitional B-cells?) or a B-cell population that develops from separate lineages or an unidentified or poorly characterized B-cell subset (human B1 cells?).

In other words, the transition from transitional B-cells into the various B-cell subsets (follicular, MZ, and after antigen encounter to MBCs) is a complicated process, in which many factors are confounded, not corresponding to the ideal conditions to generate a malignant B-cell population with a homogeneous phenotype and GEP. Recent data show that the transitional B-cells present a unique heavy chain Ig repertoire different from that of pre-B cells, immature B-cells but also naïve B-cells and that is outside the trajectory of gene loss/gain between pre-B and naive stages (26). Transitional B cells are thought to contain cells other than those that are part of the pre-B → immature → transitional → naive development pathway (26). Furthermore, as discussed above, a subset of transitional B-cells can also present the functional capacity of CLL B-cells, making them an ideal candidate. Transformation could also occur during TLR9 activation of transitional B-cells. As CpG activation of these cells has been shown to lead to their differentiation into three different subsets: ASPCs that produce IgM and switched Ig isotypes, naive B-cells but also somatically mutated IgM+ IgD+ CD27+ MBCs (111). In this configuration, unmutated CLL B-cells could derive from early activated transitional B-cells and mutated CLL B-cells could derive from IgM+ IgD+ CD27+ MBCs. Based on all of the above, CD27+ transitional B-cells could be proposed as a potential normal counterpart of CLL B-cells (Figure 2).

IgM MBCs can also be considered as potential normal counterpart, as they present similar functional characteristics. An alternative possibility would be that CLL B-cells originate from virtually all B-cell subsets ranging between transitional and MBCs. In both cases, transformation occurs while the cells are responding to a particular (auto)-Ag in a T-dependent or -independent manner. As discussed above, TI Ag or T-dependent responses are able to induce SHM (and CSR) in the various B-cell subsets. When SHM occurs, the polyreactivity of the BCR of the transformed cells will be modified or lost (giving rise to mutated CLL) and when SHM does not occur the polyreactivity of the BCR is preserved (giving rise to unmutated CLL). This factor determines the susceptibility of leukemic cells to *in vivo* stimulation by Ags (exogenous, self-Ags, and environmental Ags that periodically restimulate CLL B-cells). Cells that have undergone SHM would therefore expand less rapidly and would exhibit a more benign clinical course than unmutated CLL B-cells. This view is in line with the epigenetic hypothesis of the CLL B-cell counterpart along the continuum of B-cell development and differentiation (12).

In the light of all of the above data, events occurring during terminal B-cell differentiation could be relevant to the understanding of CLL biology. Terminal differentiation induces molecular, phenotypic changes, thereby minimizing the heterogeneity of CLL B-cells and causing them to converge toward one end of the development stage, reflecting precursor B-cell features. Analysis of molecular events (including SHM, CSR, repertoire use, antibody reactivity, and differential expression of a variety of surface molecules, receptor, and transcription factors) between CLL B-cells and normal human B-cell subsets as the B-cell progresses to mature PCs in response to defined stimuli could help to define a cellular normal counterpart of malignant cells. The normal counterpart of CLL B-cells could therefore be studied *via* a functional approach. However, this type of approach will require further research and functional characterization of B-cell subsets.

## Author Contributions

WD and HG conceptualized and wrote the manuscript and created the figures. HG, BG, and J-PM revised and edited the manuscript. All authors approved the submitted final version.

## Conflict of Interest Statement

The authors declare that the research was conducted in the absence of any commercial or financial relationships that could be construed as a potential conflict of interest.

## References

[1] Kipps TJ Stevenson FK Wu CJ Croce CM Packham G Wierda WG Chronic lymphocytic leukaemia. Nat Rev Dis Primers (2017) 3:16096. 10.1038/nrdp.2016.96 28102226 PMC5336551

[2] Fabbri G Dalla-Favera R The molecular pathogenesis of chronic lymphocytic leukaemia. Nat Rev Cancer (2016) 16(3):145–62. 10.1038/nrc.2016.8 26911189

[3] Ghamlouch H Nguyen-Khac F Bernard OA. Chronic lymphocytic leukaemia genomics and the precision medicine era. Br J Haematol (2017) 178(6):852–70. 10.1111/bjh.14719 28444740

[4] Kuppers R Klein U Hansmann ML Rajewsky K Cellular origin of human B-cell lymphomas. N Engl J Med (1999) 341(20):1520–9. 10.1056/NEJM199911113412007 10559454

[5] Kuppers R Mechanisms of B-cell lymphoma pathogenesis. Nat Rev Cancer (2005) 5(4):251–62. 10.1038/nrc1589 15803153

[6] Caligaris-Cappio F Ghia P The normal counterpart to the chronic lymphocytic leukemia B cell. Best Pract Res Clin Haematol (2007) 20(3):385–97. 10.1016/j.beha.2007.02.005 17707828

[7] Chiorazzi N Ferrarini M. Cellular origin(s) of chronic lymphocytic leukemia: cautionary notes and additional considerations and possibilities. Blood (2011) 117(6):1781–91. 10.1182/blood-2010-07-155663 21148333 PMC3056635

[8] Garcia-Munoz R Galiacho VR Llorente L. Immunological aspects in chronic lymphocytic leukemia (CLL) development. Ann Hematol (2012) 91(7):981–96. 10.1007/s00277-012-1460-z 22526361 PMC3368117

[9] Negrini M Cutrona G Bassi C Fabris S Zagatti B Colombo M MicroRNAome expression in chronic lymphocytic leukemia: comparison with normal B-cell subsets and correlations with prognostic and clinical parameters. Clin Cancer Res (2014) 20(15):4141–53. 10.1158/1078-0432.CCR-13-2497 24916701

[10] Ronchetti D Manzoni M Agnelli L Vinci C Fabris S Cutrona G lncRNA profiling in early-stage chronic lymphocytic leukemia identifies transcriptional fingerprints with relevance in clinical outcome. Blood Cancer J (2016) 6(9):e468. 10.1038/bcj.2016.77 27611921 PMC5056969

[11] Kulis M Heath S Bibikova M Queiros AC Navarro A Clot G Epigenomic analysis detects widespread gene-body DNA hypomethylation in chronic lymphocytic leukemia. Nat Genet (2012) 44(11):1236–42. 10.1038/ng.2443 23064414

[12] Oakes CC Seifert M Assenov Y Gu L Przekopowitz M Ruppert AS DNA methylation dynamics during B cell maturation underlie a continuum of disease phenotypes in chronic lymphocytic leukemia. Nat Genet (2016) 48(3):253–64. 10.1038/ng.3488 26780610 PMC4963005

[13] Smith EN Ghia EM DeBoever CM Rassenti LZ Jepsen K Yoon KA Genetic and epigenetic profiling of CLL disease progression reveals limited somatic evolution and suggests a relationship to memory-cell development. Blood Cancer J (2015) 5:e303. 10.1038/bcj.2015.14 25860294 PMC4450323

[14] Ghamlouch H Ouled-Haddou H Guyart A Regnier A Trudel S Claisse JF TLR9 ligand (CpG oligodeoxynucleotide) induces CLL B-cells to differentiate into CD20(+) antibody-secreting cells. Front Immunol (2014) 5:292. 10.3389/fimmu.2014.00292 24982661 PMC4058906

[15] Oppezzo P Magnac C Bianchi S Vuillier F Tiscornia A Dumas G Do CLL B cells correspond to naive or memory B-lymphocytes? Evidence for an active Ig switch unrelated to phenotype expression and Ig mutational pattern in B-CLL cells. Leukemia (2002) 16(12):2438–46. 10.1038/sj.leu.2402731 12454750

[16] Marasco E Farroni C Cascioli S Marcellini V Scarsella M Giorda E B-cell activation with CD40L or CpG measures the function of B-cell subsets and identifies specific defects in immunodeficient patients. Eur J Immunol (2017) 47(1):131–43. 10.1002/eji.201646574 27800605

[17] Patten PE Ferrer G Chen SS Simone R Marsilio S Yan XJ Chronic lymphocytic leukemia cells diversify and differentiate in vivo via a nonclassical Th1-dependent, Bcl-6-deficient process. JCI Insight (2016) 1(4):e86288. 10.1172/jci.insight.86288 27158669 PMC4855875

[18] Gutierrez A Jr Arendt BK Tschumper RC Kay NE Zent CS Jelinek DF. Differentiation of chronic lymphocytic leukemia B cells into immunoglobulin secreting cells decreases LEF-1 expression. PLoS One (2011) 6(10):e26056. 10.1371/journal.pone.0026056 21998751 PMC3188588

[19] Hoogeboom R Reinten RJ Schot JJ Guikema JE Bende RJ van Noesel CJ In vitro induction of antibody secretion of primary B-cell chronic lymphocytic leukaemia cells. Leukemia (2015) 29(1):244–7. 10.1038/leu.2014.266 25204568

[20] Duckworth A Glenn M Slupsky JR Packham G Kalakonda N. Variable induction of PRDM1 and differentiation in chronic lymphocytic leukemia is associated with anergy. Blood (2014) 123(21):3277–85. 10.1182/blood-2013-11-539049 24637363

[21] Ghamlouch H Darwiche W Hodroge A Ouled-Haddou H Dupont S Singh AR Factors involved in CLL pathogenesis and cell survival are disrupted by differentiation of CLL B-cells into antibody-secreting cells. Oncotarget (2015) 6(21):18484–503. 10.18632/oncotarget.3941 26050196 PMC4621905

[22] van Kooten C Rensink I Aarden L van Oers R. Differentiation of purified malignant B cells induced by PMA or by activated normal T cells. Leukemia (1993) 7(10):1576–84. 8105156

[23] Fu SM Winchester RJ Feizi T Walzer PD Kunkel HG. Idiotypic specificity of surface immunoglobulin and the maturation of leukemic bone-marrow-derived lymphocytes. Proc Natl Acad Sci U S A (1974) 71(11):4487–90. 10.1073/pnas.71.11.4487 4612522 PMC433911

[24] Rudders RA Ross R. Partial characterization of the shift from IgG to IgA synthesis in the clonal differentiation of human leukemic bone marrow-derived lymphocytes. J Exp Med (1975) 142(3):549–59. 10.1084/jem.142.3.549 809529 PMC2189926

[25] Weisel F Shlomchik M. Memory B cells of mice and humans. Annu Rev Immunol (2017) 35:255–84. 10.1146/annurev-immunol-041015-055531 28142324

[26] Martin VG Wu YB Townsend CL Lu GH O’Hare JS Mozeika A Transitional B cells in early human B cell development – time to revisit the paradigm? Front Immunol (2016) 7:546. 10.3389/fimmu.2016.00546 27994589 PMC5133252

[27] Sabouri Z Perotti S Spierings E Humburg P Yabas M Bergmann H IgD attenuates the IgM-induced anergy response in transitional and mature B cells. Nat Commun (2016) 7:13381. 10.1038/ncomms13381 27830696 PMC5109548

[28] Sims GP Ettinger R Shirota Y Yarboro CH Illei GG Lipsky PE. Identification and characterization of circulating human transitional B cells. Blood (2005) 105(11):4390–8. 10.1182/blood-2004-11-4284 15701725 PMC1895038

[29] Cerutti A Cols M Puga I. Marginal zone B cells: virtues of innate-like antibody-producing lymphocytes. Nat Rev Immunol (2013) 13(2):118–32. 10.1038/nri3383 23348416 PMC3652659

[30] Lee J Kuchen S Fischer R Chang S Lipsky PE. Identification and characterization of a human CD5+ pre-naive B cell population. J Immunol (2009) 182(7):4116–26. 10.4049/jimmunol.0803391 19299709

[31] Suryani S Fulcher DA Santner-Nanan B Nanan R Wong M Shaw PJ Differential expression of CD21 identifies developmentally and functionally distinct subsets of human transitional B cells. Blood (2010) 115(3):519–29. 10.1182/blood-2009-07-234799 19965666

[32] Seifert M Sellmann L Bloehdorn J Wein F Stilgenbauer S Durig J Cellular origin and pathophysiology of chronic lymphocytic leukemia. J Exp Med (2012) 209(12):2183–98. 10.1084/jem.20120833 23091163 PMC3501361

[33] Goodnow CC Vinuesa CG Randall KL Mackay F Brink R. Control systems and decision making for antibody production. Nat Immunol (2010) 11(8):681–8. 10.1038/ni.1900 20644574

[34] MacLennan IC Toellner KM Cunningham AF Serre K Sze DM Zuniga E Extrafollicular antibody responses. Immunol Rev (2003) 194:8–18. 10.1034/j.1600-065X.2003.00058.x 12846803

[35] Martin F Oliver AM Kearney JF. Marginal zone and B1 B cells unite in the early response against T-independent blood-borne particulate antigens. Immunity (2001) 14(5):617–29. 10.1016/S1074-7613(01)00129-7 11371363

[36] Di Sabatino A Carsetti R Corazza GR. Post-splenectomy and hyposplenic states. Lancet (2011) 378(9785):86–97. 10.1016/S0140-6736(10)61493-6 21474172

[37] Seifert M Kuppers R. Human memory B cells. Leukemia (2016) 30(12):2283–92. 10.1038/leu.2016.226 27499139

[38] Seifert M Przekopowitz M Taudien S Lollies A Ronge V Drees B Functional capacities of human IgM memory B cells in early inflammatory responses and secondary germinal center reactions. Proc Natl Acad Sci U S A (2015) 112(6):E546–55. 10.1073/pnas.1416276112 25624468 PMC4330750

[39] Weller S Braun MC Tan BK Rosenwald A Cordier C Conley ME Human blood IgM “memory” B cells are circulating splenic marginal zone B cells harboring a prediversified immunoglobulin repertoire. Blood (2004) 104(12):3647–54. 10.1182/blood-2004-01-0346 15191950 PMC2590648

[40] Weill JC Weller S Reynaud CA. Human marginal zone B cells. Annu Rev Immunol (2009) 27:267–85. 10.1146/annurev.immunol.021908.132607 19302041

[41] Bagnara D Squillario M Kipling D Mora T Walczak AM Da Silva L A reassessment of IgM memory subsets in humans. J Immunol (2015) 195(8):3716–24. 10.4049/jimmunol.1500753 26355154 PMC4594759

[42] Sindhava VJ Bondada S. Multiple regulatory mechanisms control B-1 B cell activation. Front Immunol (2012) 3:372. 10.3389/fimmu.2012.00372 23251136 PMC3523257

[43] Haas KM. B-1 lymphocytes in mice and nonhuman primates. Ann N Y Acad Sci (2015) 1362:98–109. 10.1111/nyas.12760 25930711 PMC4627897

[44] Casali P Burastero SE Nakamura M Inghirami G Notkins AL. Human lymphocytes making rheumatoid factor and antibody to ssDNA belong to Leu-1+ B-cell subset. Science (1987) 236(4797):77–81. 10.1126/science.3105056 3105056

[45] Hardy RR Hayakawa K Shimizu M Yamasaki K Kishimoto T. Rheumatoid factor secretion from human Leu-1+ B cells. Science (1987) 236(4797):81–3. 10.1126/science.3105057 3105057

[46] Simon Q Pers JO Cornec D Le Pottier L Mageed RA Hillion S In-depth characterization of CD24(high)CD38(high) transitional human B cells reveals different regulatory profiles. J Allergy Clin Immunol (2016) 137(5):1577–84.e10. 10.1016/j.jaci.2015.09.014 26525227

[47] Griffin DO Holodick NE Rothstein TL Human B1 cells in umbilical cord and adult peripheral blood express the novel phenotype CD20+ CD27+ CD43+ CD70. J Exp Med (2011) 208(1):67–80. 10.1084/jem.201014992011113c 21220451 PMC3023138

[48] Candando KM Lykken JM Tedder TF. B10 cell regulation of health and disease. Immunol Rev (2014) 259(1):259–72. 10.1111/imr.12176 24712471 PMC4049540

[49] Quach TD Rodriguez-Zhurbenko N Hopkins TJ Guo X Hernandez AM Li W Distinctions among circulating antibody-secreting cell populations, including B-1 cells, in human adult peripheral blood. J Immunol (2016) 196(3):1060–9. 10.4049/jimmunol.1501843 26740107 PMC5351554

[50] Covens K Verbinnen B Geukens N Meyts I Schuit F Van Lommel L Characterization of proposed human B-1 cells reveals pre-plasmablast phenotype. Blood (2013) 121(26):5176–83. 10.1182/blood-2012-12-471953 23613519

[51] Reynaud CA Weill JC Gene profiling of CD11b(+) and CD11b(-) B1 cell subsets reveals potential cell sorting artifacts. J Exp Med (2012) 209(3):433–4. 10.1084/jem.20120402 22412175 PMC3302223

[52] Bhat NM Kantor AB Bieber MM Stall AM Herzenberg LA Teng NN. The ontogeny and functional characteristics of human B-1 (CD5+ B) cells. Int Immunol (1992) 4(2):243–52. 10.1093/intimm/4.2.243 1377947

[53] Hsu MC Toellner KM Vinuesa CG Maclennan IC. B cell clones that sustain long-term plasmablast growth in T-independent extrafollicular antibody responses. Proc Natl Acad Sci U S A (2006) 103(15):5905–10. 10.1073/pnas.0601502103 16585532 PMC1424660

[54] Alugupalli KR Leong JM Woodland RT Muramatsu M Honjo T Gerstein RM. B1b lymphocytes confer T cell-independent long-lasting immunity. Immunity (2004) 21(3):379–90. 10.1016/j.immuni.2004.06.019 15357949

[55] Baumgarth N. B-1 cell heterogeneity and the regulation of natural and antigen-induced IgM production. Front Immunol (2016) 7:324. 10.3389/fimmu.2016.00324 27667991 PMC5016532

[56] Yang Y Ghosn EE Cole LE Obukhanych TV Sadate-Ngatchou P Vogel SN Antigen-specific memory in B-1a and its relationship to natural immunity. Proc Natl Acad Sci U S A (2012) 109(14):5388–93. 10.1073/pnas.1121627109 22421135 PMC3325686

[57] Maseda D Smith SH DiLillo DJ Bryant JM Candando KM Weaver CT Regulatory B10 cells differentiate into antibody-secreting cells after transient IL-10 production in vivo. J Immunol (2012) 188(3):1036–48. 10.4049/jimmunol.1102500 22198952 PMC3262922

[58] Manz RA Hauser AE Hiepe F Radbruch A. Maintenance of serum antibody levels. Annu Rev Immunol (2005) 23:367–86. 10.1146/annurev.immunol.23.021704.115723 15771575

[59] Bernasconi NL Traggiai E Lanzavecchia A. Maintenance of serological memory by polyclonal activation of human memory B cells. Science (2002) 298(5601):2199–202. 10.1126/science.1076071 12481138

[60] Reynaud CA Descatoire M Dogan I Huetz F Weller S Weill JC. IgM memory B cells: a mouse/human paradox. Cell Mol Life Sci (2012) 69(10):1625–34. 10.1007/s00018-012-0971-z 22481437 PMC3337997

[61] Capolunghi F Rosado MM Sinibaldi M Aranburu A Carsetti R. Why do we need IgM memory B cells? Immunol Lett (2013) 152(2):114–20. 10.1016/j.imlet.2013.04.007 23660557

[62] Kurosaki T Kometani K Ise W Memory B cells. Nat Rev Immunol (2015) 15(3):149–59. 10.1038/nri3802 25677494

[63] Dogan I Bertocci B Vilmont V Delbos F Megret J Storck S Multiple layers of B cell memory with different effector functions. Nat Immunol (2009) 10(12):1292–9. 10.1038/ni.1814 19855380

[64] Pape KA Taylor JJ Maul RW Gearhart PJ Jenkins MK. Different B cell populations mediate early and late memory during an endogenous immune response. Science (2011) 331(6021):1203–7. 10.1126/science.1201730 21310965 PMC3993090

[65] Rubtsova K Rubtsov AV Cancro MP Marrack P. Age-associated B cells: a T-bet-dependent effector with roles in protective and pathogenic immunity. J Immunol (2015) 195(5):1933–7. 10.4049/jimmunol.1501209 26297793 PMC4548292

[66] Knox JJ Buggert M Kardava L Seaton KE Eller MA Canaday DH T-bet+ B cells are induced by human viral infections and dominate the HIV gp140 response. JCI Insight (2017) 2(8):92943. 10.1172/jci.insight.92943 28422752 PMC5396521

[67] Portugal S Obeng-Adjei N Moir S Crompton PD Pierce SK. Atypical memory B cells in human chronic infectious diseases: an interim report. Cell Immunol (2017) 321:18–25. 10.1016/j.cellimm.2017.07.003 28735813 PMC5732066

[68] Carsetti R Rosado MM Wardmann H. Peripheral development of B cells in mouse and man. Immunol Rev (2004) 197:179–91. 10.1111/j.0105-2896.2004.0109.x 14962195

[69] Kuraoka M Liao D Yang K Allgood SD Levesque MC Kelsoe G Activation-induced cytidine deaminase expression and activity in the absence of germinal centers: insights into hyper-IgM syndrome. J Immunol (2009) 183(5):3237–48. 10.4049/jimmunol.0901548 19667096 PMC2779701

[70] Taylor JJ Pape KA Jenkins MK. A germinal center-independent pathway generates unswitched memory B cells early in the primary response. J Exp Med (2012) 209(3):597–606. 10.1084/jem.20111696 22370719 PMC3302224

[71] Berkowska MA Driessen GJ Bikos V Grosserichter-Wagener C Stamatopoulos K Cerutti A Human memory B cells originate from three distinct germinal center-dependent and -independent maturation pathways. Blood (2011) 118(8):2150–8. 10.1182/blood-2011-04-345579 21690558 PMC3342861

[72] Aranburu A Piano Mortari E Baban A Giorda E Cascioli S Marcellini V Human B-cell memory is shaped by age- and tissue-specific T-independent and GC-dependent events. Eur J Immunol (2017) 47(2):327–44. 10.1002/eji.201646642 27859047

[73] Weller S Mamani-Matsuda M Picard C Cordier C Lecoeuche D Gauthier F Somatic diversification in the absence of antigen-driven responses is the hallmark of the IgM+ IgD+ CD27+ B cell repertoire in infants. J Exp Med (2008) 205(6):1331–42. 10.1084/jem.20071555 18519648 PMC2413031

[74] Wardemann H Yurasov S Schaefer A Young JW Meffre E Nussenzweig MC. Predominant autoantibody production by early human B cell precursors. Science (2003) 301(5638):1374–7. 10.1126/science.1086907 12920303

[75] Tsuiji M Yurasov S Velinzon K Thomas S Nussenzweig MC Wardemann H. A checkpoint for autoreactivity in human IgM+ memory B cell development. J Exp Med (2006) 203(2):393–400. 10.1084/jem.20052033 16446381 PMC2118214

[76] Tiller T Tsuiji M Yurasov S Velinzon K Nussenzweig MC Wardemann H. Autoreactivity in human IgG+ memory B cells. Immunity (2007) 26(2):205–13. 10.1016/j.immuni.2007.01.009 17306569 PMC1839941

[77] Koelsch K Zheng NY Zhang Q Duty A Helms C Mathias MD Mature B cells class switched to IgD are autoreactive in healthy individuals. J Clin Invest (2007) 117(6):1558–65. 10.1172/JCI27628 17510706 PMC1866247

[78] Scheid JF Mouquet H Kofer J Yurasov S Nussenzweig MC Wardemann H. Differential regulation of self-reactivity discriminates between IgG+ human circulating memory B cells and bone marrow plasma cells. Proc Natl Acad Sci U S A (2011) 108(44):18044–8. 10.1073/pnas.1113395108 22025722 PMC3207704

[79] Nutt SL Hodgkin PD Tarlinton DM Corcoran LM The generation of antibody-secreting plasma cells. Nat Rev Immunol (2015) 15(3):160–71. 10.1038/nri3795 25698678

[80] Fagarasan S Honjo T. T-independent immune response: new aspects of B cell biology. Science (2000) 290(5489):89–92. 10.1126/science.290.5489.89 11021805

[81] Vos Q Lees A Wu ZQ Snapper CM Mond JJ. B-cell activation by T-cell-independent type 2 antigens as an integral part of the humoral immune response to pathogenic microorganisms. Immunol Rev (2000) 176:154–70. 10.1034/j.1600-065X.2000.00607.x 11043775

[82] Pereira JP Kelly LM Xu Y Cyster JG. EBI2 mediates B cell segregation between the outer and centre follicle. Nature (2009) 460(7259):1122–6. 10.1038/nature08226 19597478 PMC2809436

[83] Green JA Suzuki K Cho B Willison LD Palmer D Allen CD The sphingosine 1-phosphate receptor S1P(2) maintains the homeostasis of germinal center B cells and promotes niche confinement. Nat Immunol (2011) 12(7):672–80. 10.1038/ni.2047 21642988 PMC3158008

[84] Di Niro R Lee SJ Vander Heiden JA Elsner RA Trivedi N Bannock JM Salmonella infection drives promiscuous B cell activation followed by extrafollicular affinity maturation. Immunity (2015) 43(1):120–31. 10.1016/j.immuni.2015.06.013 26187411 PMC4523395

[85] Bohannon C Powers R Satyabhama L Cui A Tipton C Michaeli M Long-lived antigen-induced IgM plasma cells demonstrate somatic mutations and contribute to long-term protection. Nat Commun (2016) 7:11826. 10.1038/ncomms11826 27270306 PMC4899631

[86] Miyauchi K Sugimoto-Ishige A Harada Y Adachi Y Usami Y Kaji T Protective neutralizing influenza antibody response in the absence of T follicular helper cells. Nat Immunol (2016) 17(12):1447–58. 10.1038/ni.3563 27798619

[87] Lee SK Rigby RJ Zotos D Tsai LM Kawamoto S Marshall JL B cell priming for extrafollicular antibody responses requires Bcl-6 expression by T cells. J Exp Med (2011) 208(7):1377–88. 10.1084/jem.20102065 21708925 PMC3135363

[88] Oestreich KJ Mohn SE Weinmann AS. Molecular mechanisms that control the expression and activity of Bcl-6 in TH1 cells to regulate flexibility with a TFH-like gene profile. Nat Immunol (2012) 13(4):405–11. 10.1038/ni.2242 22406686 PMC3561768

[89] Bortnick A Chernova I Quinn WJ III Mugnier M Cancro MP Allman D. Long-lived bone marrow plasma cells are induced early in response to T cell-independent or T cell-dependent antigens. J Immunol (2012) 188(11):5389–96. 10.4049/jimmunol.1102808 22529295 PMC4341991

[90] Taillardet M Haffar G Mondiere P Asensio MJ Gheit H Burdin N The thymus-independent immunity conferred by a pneumococcal polysaccharide is mediated by long-lived plasma cells. Blood (2009) 114(20):4432–40. 10.1182/blood-2009-01-200014 19767510

[91] Onodera T Takahashi Y Yokoi Y Ato M Kodama Y Hachimura S Memory B cells in the lung participate in protective humoral immune responses to pulmonary influenza virus reinfection. Proc Natl Acad Sci U S A (2012) 109(7):2485–90. 10.1073/pnas.1115369109 22308386 PMC3289300

[92] Pellat-Deceunynck C Defrance T The origin of the plasma-cell heterogeneity. Front Immunol (2015) 6:5 10.3389/fimmu.2015.00005 25667588 PMC4304349

[93] Medina F Segundo C Campos-Caro A Gonzalez-Garcia I Brieva JA. The heterogeneity shown by human plasma cells from tonsil, blood, and bone marrow reveals graded stages of increasing maturity, but local profiles of adhesion molecule expression. Blood (2002) 99(6):2154–61. 10.1182/blood.V99.6.2154 11877292

[94] Halliley JL Tipton CM Liesveld J Rosenberg AF Darce J Gregoretti IV Long-lived plasma cells are contained within the CD19(-)CD38(hi)CD138(+) subset in human bone marrow. Immunity (2015) 43(1):132–45. 10.1016/j.immuni.2015.06.016 26187412 PMC4680845

[95] Perez ME Billordo LA Baz P Fainboim L Arana E. Human memory B cells isolated from blood and tonsils are functionally distinctive. Immunol Cell Biol (2014) 92(10):882–7. 10.1038/icb.2014.59 25047642

[96] Moens L Tangye SG. Cytokine-mediated regulation of plasma cell generation: IL-21 takes center stage. Front Immunol (2014) 5:65. 10.3389/fimmu.2014.00065 24600453 PMC3927127

[97] Arpin C Dechanet J Van Kooten C Merville P Grouard G Briere F Generation of memory B cells and plasma cells in vitro. Science (1995) 268(5211):720–2. 10.1126/science.7537388 7537388

[98] Liu YJ Banchereau J. Regulation of B-cell commitment to plasma cells or to memory B cells. Semin Immunol (1997) 9(4):235–40. 10.1006/smim.1997.0080 9237929

[99] Tarte K De Vos J Thykjaer T Zhan F Fiol G Costes V Generation of polyclonal plasmablasts from peripheral blood B cells: a normal counterpart of malignant plasmablasts. Blood (2002) 100(4):1113–22. 12149187

[100] Macallan DC Wallace DL Zhang Y Ghattas H Asquith B de Lara C B-cell kinetics in humans: rapid turnover of peripheral blood memory cells. Blood (2005) 105(9):3633–40. 10.1182/blood-2004-09-3740 15644412

[101] Shi Y Agematsu K Ochs HD Sugane K. Functional analysis of human memory B-cell subpopulations: IgD+CD27+ B cells are crucial in secondary immune response by producing high affinity IgM. Clin Immunol (2003) 108(2):128–37. 10.1016/S1521-6616(03)00092-5 12921759

[102] Werner-Favre C Bovia F Schneider P Holler N Barnet M Kindler V IgG subclass switch capacity is low in switched and in IgM-only, but high in IgD+IgM+, post-germinal center (CD27+) human B cells. Eur J Immunol (2001) 31(1):243–9. 10.1002/1521-4141(200101)31:1<243::AID-IMMU243>3.0.CO;2-0 11265640

[103] Fecteau JF Neron S. CD40 stimulation of human peripheral B lymphocytes: distinct response from naive and memory cells. J Immunol (2003) 171(9):4621–9. 10.4049/jimmunol.171.9.4621 14568936

[104] Kindler V Zubler RH. Memory, but not naive, peripheral blood B lymphocytes differentiate into Ig-secreting cells after CD40 ligation and costimulation with IL-4 and the differentiation factors IL-2, IL-10, and IL-3. J Immunol (1997) 159(5):2085–90. 9278293

[105] Vasquez C Franco MA Angel J. Rapid proliferation and differentiation of a subset of circulating IgM memory B cells to a CpG/cytokine stimulus in vitro. PLoS One (2015) 10(10):e0139718. 10.1371/journal.pone.0139718 26439739 PMC4595470

[106] Geffroy-Luseau A Chiron D Descamps G Jego G Amiot M Pellat-Deceunynck C. TLR9 ligand induces the generation of CD20+ plasmablasts and plasma cells from CD27+ memory B-cells. Front Immunol (2011) 2:83. 10.3389/fimmu.2011.00083 22566872 PMC3342082

[107] Dugas-Bourdages E Neron S Roy A Darveau A Delage R. Persistent polyclonal B cell lymphocytosis B cells can be activated through CD40-CD154 interaction. Adv Hematol (2014) 2014:854124. 10.1155/2014/854124 25580126 PMC4279877

[108] Jourdan M Caraux A De Vos J Fiol G Larroque M Cognot C An in vitro model of differentiation of memory B cells into plasmablasts and plasma cells including detailed phenotypic and molecular characterization. Blood (2009) 114(25):5173–81. 10.1182/blood-2009-07-235960 19846886 PMC2834398

[109] Simchoni N Cunningham-Rundles C. TLR7- and TLR9-responsive human B cells share phenotypic and genetic characteristics. J Immunol (2015) 194(7):3035–44. 10.4049/jimmunol.1402690 25740945 PMC4369401

[110] Bekeredjian-Ding I Doster A Schiller M Heyder P Lorenz HM Schraven B TLR9-activating DNA up-regulates ZAP70 via sustained PKB induction in IgM+ B cells. J Immunol (2008) 181(12):8267–77. 10.4049/jimmunol.181.12.8267 19050243

[111] Capolunghi F Cascioli S Giorda E Rosado MM Plebani A Auriti C CpG drives human transitional B cells to terminal differentiation and production of natural antibodies. J Immunol (2008) 180(2):800–8. 10.4049/jimmunol.180.2.800 18178818

[112] Bekeredjian-Ding IB Wagner M Hornung V Giese T Schnurr M Endres S Plasmacytoid dendritic cells control TLR7 sensitivity of naive B cells via type I IFN. J Immunol (2005) 174(7):4043–50. 10.4049/jimmunol.174.7.4043 15778362

[113] Poeck H Wagner M Battiany J Rothenfusser S Wellisch D Hornung V Plasmacytoid dendritic cells, antigen, and CpG-C license human B cells for plasma cell differentiation and immunoglobulin production in the absence of T-cell help. Blood (2004) 103(8):3058–64. 10.1182/blood-2003-08-2972 15070685

[114] Bernasconi NL Onai N Lanzavecchia A. A role for toll-like receptors in acquired immunity: up-regulation of TLR9 by BCR triggering in naive B cells and constitutive expression in memory B cells. Blood (2003) 101(11):4500–4. 10.1182/blood-2002-11-3569 12560217

[115] Le Gallou S Caron G Delaloy C Rossille D Tarte K Fest T. IL-2 requirement for human plasma cell generation: coupling differentiation and proliferation by enhancing MAPK-ERK signaling. J Immunol (2012) 189(1):161–73. 10.4049/jimmunol.1200301 22634617

[116] Giordani L Sanchez M Libri I Quaranta MG Mattioli B Viora M. IFN-alpha amplifies human naive B cell TLR-9-mediated activation and Ig production. J Leukoc Biol (2009) 86(2):261–71. 10.1189/jlb.0908560 19401392

[117] Huggins J Pellegrin T Felgar RE Wei C Brown M Zheng B CpG DNA activation and plasma-cell differentiation of CD27- naive human B cells. Blood (2007) 109(4):1611–9. 10.1182/blood-2006-03-008441 17032927 PMC1794051

[118] Ruprecht CR Lanzavecchia A. Toll-like receptor stimulation as a third signal required for activation of human naive B cells. Eur J Immunol (2006) 36(4):810–6. 10.1002/eji.200535744 16541472

[119] Jiang W Lederman MM Harding CV Rodriguez B Mohner RJ Sieg SF TLR9 stimulation drives naive B cells to proliferate and to attain enhanced antigen presenting function. Eur J Immunol (2007) 37(8):2205–13. 10.1002/eji.200636984 17621369

[120] Glaum MC Narula S Song D Zheng Y Anderson AL Pletcher CH Toll-like receptor 7-induced naive human B-cell differentiation and immunoglobulin production. J Allergy Clin Immunol (2009) 123(1):224–30.e4. 10.1016/j.jaci.2008.09.018 18995892

[121] Li FJ Schreeder DM Li R Wu J Davis RS. FCRL3 promotes TLR9-induced B-cell activation and suppresses plasma cell differentiation. Eur J Immunol (2013) 43(11):2980–92. 10.1002/eji.201243068 23857366 PMC3838486

[122] Dubois B Massacrier C Vanbervliet B Fayette J Briere F Banchereau J Critical role of IL-12 in dendritic cell-induced differentiation of naive B lymphocytes. J Immunol (1998) 161(5):2223–31. 9725215

[123] Arpin C Banchereau J Liu YJ. Memory B cells are biased towards terminal differentiation: a strategy that may prevent repertoire freezing. J Exp Med (1997) 186(6):931–40. 10.1084/jem.186.6.931 9294147 PMC2199043

[124] Guerrier T Youinou P Pers JO Jamin C. TLR9 drives the development of transitional B cells towards the marginal zone pathway and promotes autoimmunity. J Autoimmun (2012) 39(3):173–9. 10.1016/j.jaut.2012.05.012 22695187

[125] Tangye SG Avery DT Hodgkin PD. A division-linked mechanism for the rapid generation of Ig-secreting cells from human memory B cells. J Immunol (2003) 170(1):261–9. 10.4049/jimmunol.170.1.261 12496408

[126] Avery DT Ellyard JI Mackay F Corcoran LM Hodgkin PD Tangye SG. Increased expression of CD27 on activated human memory B cells correlates with their commitment to the plasma cell lineage. J Immunol (2005) 174(7):4034–42. 10.4049/jimmunol.174.7.4034 15778361

[127] Moens L Kane A Tangye SG Naive and memory B cells exhibit distinct biochemical responses following BCR engagement. Immunol Cell Biol (2016) 94(8):774–86. 10.1038/icb.2016.41 27101923

[128] Suzuki N Sakane T. Induction of excessive B cell proliferation and differentiation by an in vitro stimulus in culture in human systemic lupus erythematosus. J Clin Invest (1989) 83(3):937–44. 10.1172/JCI113979 2646322 PMC303769

[129] Banchereau J Rousset F Human B lymphocytes: phenotype, proliferation, and differentiation. Adv Immunol (1992) 52:125–262. 10.1016/S0065-2776(08)60876-7 1442306

[130] Tangye SG Avery DT Deenick EK Hodgkin PD. Intrinsic differences in the proliferation of naive and memory human B cells as a mechanism for enhanced secondary immune responses. J Immunol (2003) 170(2):686–94. 10.4049/jimmunol.170.2.686 12517929

[131] Deenick EK Avery DT Chan A Berglund LJ Ives ML Moens L Naive and memory human B cells have distinct requirements for STAT3 activation to differentiate into antibody-secreting plasma cells. J Exp Med (2013) 210(12):2739–53. 10.1084/jem.20130323 24218138 PMC3832925

[132] Good KL Avery DT Tangye SG. Resting human memory B cells are intrinsically programmed for enhanced survival and responsiveness to diverse stimuli compared to naive B cells. J Immunol (2009) 182(2):890–901. 10.4049/jimmunol.182.2.890 19124732

[133] Liu YJ Barthelemy C de Bouteiller O Arpin C Durand I Banchereau J. Memory B cells from human tonsils colonize mucosal epithelium and directly present antigen to T cells by rapid up-regulation of B7-1 and B7-2. Immunity (1995) 2(3):239–48. 10.1016/1074-7613(95)90048-9 7535180

[134] Neron S Nadeau PJ Darveau A Leblanc JF. Tuning of CD40-CD154 interactions in human B-lymphocyte activation: a broad array of in vitro models for a complex in vivo situation. Arch Immunol Ther Exp (2011) 59(1):25–40. 10.1007/s00005-010-0108-8 21234809

[135] Maurer D Fischer GF Fae I Majdic O Stuhlmeier K Von Jeney N IgM and IgG but not cytokine secretion is restricted to the CD27+ B lymphocyte subset. J Immunol (1992) 148(12):3700–5. 1318333

[136] Agematsu K Nagumo H Yang FC Nakazawa T Fukushima K Ito S B cell subpopulations separated by CD27 and crucial collaboration of CD27+ B cells and helper T cells in immunoglobulin production. Eur J Immunol (1997) 27(8):2073–9. 10.1002/eji.1830270835 9295047

[137] Cantaert T Schickel JN Bannock JM Ng YS Massad C Oe T Activation-induced cytidine deaminase expression in human B cell precursors is essential for central B cell tolerance. Immunity (2015) 43(5):884–95. 10.1016/j.immuni.2015.10.002 26546282 PMC4654975

[138] Kuraoka M Snowden PB Nojima T Verkoczy L Haynes BF Kitamura D BCR and endosomal TLR signals synergize to increase AID expression and establish central B cell tolerance. Cell Rep (2017) 18(7):1627–35. 10.1016/j.celrep.2017.01.050 28199836 PMC5328188

[139] Aranburu A Ceccarelli S Giorda E Lasorella R Ballatore G Carsetti R. TLR ligation triggers somatic hypermutation in transitional B cells inducing the generation of IgM memory B cells. J Immunol (2010) 185(12):7293–301. 10.4049/jimmunol.1002722 21078901

[140] Ueda Y Liao D Yang K Patel A Kelsoe G. T-independent activation-induced cytidine deaminase expression, class-switch recombination, and antibody production by immature/transitional 1 B cells. J Immunol (2007) 178(6):3593–601. 10.4049/jimmunol.178.6.3593 17339456 PMC1955467

[141] Hasan M Lopez-Herrera G Blomberg KE Lindvall JM Berglof A Smith CI Defective toll-like receptor 9-mediated cytokine production in B cells from Bruton’s tyrosine kinase-deficient mice. Immunology (2008) 123(2):239–49. 10.1111/j.1365-2567.2007.02693.x 17725607 PMC2433303

[142] Nawata Y Stall AM Herzenberg LA Eugui EM Allison AC. Surface immunoglobulin ligands and cytokines differentially affect proliferation and antibody production by human CD5+ and CD5- B lymphocytes. Int Immunol (1990) 2(7):603–14. 10.1093/intimm/2.7.603 1703783

[143] Zupo S Dono M Azzoni L Chiorazzi N Ferrarini M. Evidence for differential responsiveness of human CD5+ and CD5- B cell subsets to T cell-independent mitogens. Eur J Immunol (1991) 21(2):351–9. 10.1002/eji.1830210216 1705511

[144] Defrance T Vanbervliet B Durand I Briolay J Banchereau J. Proliferation and differentiation of human CD5+ and CD5- B cell subsets activated through their antigen receptors or CD40 antigens. Eur J Immunol (1992) 22(11):2831–9. 10.1002/eji.1830221112 1385152

[145] Durandy A Thuillier L Forveille M Fischer A. Phenotypic and functional characteristics of human newborns’ B lymphocytes. J Immunol (1990) 144(1):60–5. 1688576

[146] Punnonen J Aversa GG Vandekerckhove B Roncarolo MG de Vries JE. Induction of isotype switching and Ig production by CD5+ and CD10+ human fetal B cells. J Immunol (1992) 148(11):3398–404. 1375243

[147] Solvason N Lehuen A Kearney JF. An embryonic source of Ly1 but not conventional B cells. Int Immunol (1991) 3(6):543–50. 10.1093/intimm/3.6.543 1679664

[148] Tarlinton D B-cell differentiation in the bone marrow and the periphery. Immunol Rev (1994) 137:203–29. 10.1111/j.1600-065X.1994.tb00666.x 8034336

[149] Sanz I Wei C Lee FE Anolik J. Phenotypic and functional heterogeneity of human memory B cells. Semin Immunol (2008) 20(1):67–82. 10.1016/j.smim.2007.12.006 18258454 PMC2440717

[150] Agematsu K Nagumo H Shinozaki K Hokibara S Yasui K Terada K Absence of IgD-CD27(+) memory B cell population in X-linked hyper-IgM syndrome. J Clin Invest (1998) 102(4):853–60. 10.1172/JCI3409 9710455 PMC508949

[151] Durandy A Hivroz C Mazerolles F Schiff C Bernard F Jouanguy E Abnormal CD40-mediated activation pathway in B lymphocytes from patients with hyper-IgM syndrome and normal CD40 ligand expression. J Immunol (1997) 158(6):2576–84. 9058789

[152] Engels N Konig LM Heemann C Lutz J Tsubata T Griep S Recruitment of the cytoplasmic adaptor Grb2 to surface IgG and IgE provides antigen receptor-intrinsic costimulation to class-switched B cells. Nat Immunol (2009) 10(9):1018–25. 10.1038/ni.1764 19668218

[153] Martin SW Goodnow CC. Burst-enhancing role of the IgG membrane tail as a molecular determinant of memory. Nat Immunol (2002) 3(2):182–8. 10.1038/ni752 11812996

[154] Wu YC Kipling D Dunn-Walters DK. The relationship between CD27 negative and positive B cell populations in human peripheral blood. Front Immunol (2011) 2:81. 10.3389/fimmu.2011.00081 22566870 PMC3341955

[155] Matutes E Polliack A. Morphological and immunophenotypic features of chronic lymphocytic leukemia. Rev Clin Exp Hematol (2000) 4(1):22–47. 10.1046/j.1468-0734.2000.00002.x 11486329

[156] Oscier D Else M Matutes E Morilla R Strefford JC Catovsky D. The morphology of CLL revisited: the clinical significance of prolymphocytes and correlations with prognostic/molecular markers in the LRF CLL4 trial. Br J Haematol (2016) 174(5):767–75. 10.1111/bjh.14132 27151266 PMC4995732

[157] Baldini L Cro L Cortelezzi A Calori R Nobili L Maiolo AT Immunophenotypes in “classical” B-cell chronic lymphocytic leukemia. Correlation with normal cellular counterpart and clinical findings. Cancer (1990) 66(8):1738–42. 10.1002/1097-0142(19901015)66:8<1738::AID-CNCR2820660816>3.0.CO;2-L 2208028

[158] Damle RN Ghiotto F Valetto A Albesiano E Fais F Yan XJ B-cell chronic lymphocytic leukemia cells express a surface membrane phenotype of activated, antigen-experienced B lymphocytes. Blood (2002) 99(11):4087–93. 10.1182/blood.V99.11.4087 12010811

[159] Tangye SG Liu YJ Aversa G Phillips JH de Vries JE. Identification of functional human splenic memory B cells by expression of CD148 and CD27. J Exp Med (1998) 188(9):1691–703. 10.1084/jem.188.9.1691 9802981 PMC2212517

[160] Pascual V Liu YJ Magalski A de Bouteiller O Banchereau J Capra JD. Analysis of somatic mutation in five B cell subsets of human tonsil. J Exp Med (1994) 180(1):329–39. 10.1084/jem.180.1.329 8006591 PMC2191579

[161] Gary-Gouy H Harriague J Bismuth G Platzer C Schmitt C Dalloul AH. Human CD5 promotes B-cell survival through stimulation of autocrine IL-10 production. Blood (2002) 100(13):4537–43. 10.1182/blood-2002-05-1525 12393419

[162] Garaud S Morva A Lemoine S Hillion S Bordron A Pers JO CD5 promotes IL-10 production in chronic lymphocytic leukemia B cells through STAT3 and NFAT2 activation. J Immunol (2011) 186(8):4835–44. 10.4049/jimmunol.1003050 21398617

[163] Hillion S Saraux A Youinou P Jamin C. Expression of RAGs in peripheral B cells outside germinal centers is associated with the expression of CD5. J Immunol (2005) 174(9):5553–61. 10.4049/jimmunol.174.9.5553 15843554

[164] Harindranath N Goldfarb IS Ikematsu H Burastero SE Wilder RL Notkins AL Complete sequence of the genes encoding the VH and VL regions of low- and high-affinity monoclonal IgM and IgA1 rheumatoid factors produced by CD5+ B cells from a rheumatoid arthritis patient. Int Immunol (1991) 3(9):865–75. 10.1093/intimm/3.9.865 1718404 PMC4632984

[165] Mantovani L Wilder RL Casali P. Human rheumatoid B-1a (CD5+ B) cells make somatically hypermutated high affinity IgM rheumatoid factors. J Immunol (1993) 151(1):473–88. 7686945 PMC4625548

[166] Schettino EW Chai SK Kasaian MT Schroeder HW Jr Casali P. VHDJH gene sequences and antigen reactivity of monoclonal antibodies produced by human B-1 cells: evidence for somatic selection. J Immunol (1997) 158(5):2477–89. 9037000 PMC4631314

[167] Dono M Burgio VL Colombo M Sciacchitano S Reverberi D Tarantino V CD5+ B cells with the features of subepithelial B cells found in human tonsils. Eur J Immunol (2007) 37(8):2138–47. 10.1002/eji.200636887 17615580

[168] Fischer M Klein U Kuppers R. Molecular single-cell analysis reveals that CD5-positive peripheral blood B cells in healthy humans are characterized by rearranged Vkappa genes lacking somatic mutation. J Clin Invest (1997) 100(7):1667–76. 10.1172/JCI119691 9312164 PMC508349

[169] Packham G Krysov S Allen A Savelyeva N Steele AJ Forconi F The outcome of B-cell receptor signaling in chronic lymphocytic leukemia: proliferation or anergy. Haematologica (2014) 99(7):1138–48. 10.3324/haematol.2013.098384 24986876 PMC4077074

[170] Duhren-von Minden M Ubelhart R Schneider D Wossning T Bach MP Buchner M Chronic lymphocytic leukaemia is driven by antigen-independent cell-autonomous signalling. Nature (2012) 489(7415):309–12. 10.1038/nature11309 22885698

[171] Schroeder HW Jr Dighiero G. The pathogenesis of chronic lymphocytic leukemia: analysis of the antibody repertoire. Immunol Today (1994) 15(6):288–94. 10.1016/0167-5699(94)90009-4 7520700

[172] Klein U Tu Y Stolovitzky GA Mattioli M Cattoretti G Husson H Gene expression profiling of B cell chronic lymphocytic leukemia reveals a homogeneous phenotype related to memory B cells. J Exp Med (2001) 194(11):1625–38. 10.1084/jem.194.11.1625 11733577 PMC2193527

[173] Weller S Faili A Garcia C Braun MC Le Deist FF de Saint Basile GG CD40-CD40L independent Ig gene hypermutation suggests a second B cell diversification pathway in humans. Proc Natl Acad Sci U S A (2001) 98(3):1166–70. 10.1073/pnas.98.3.1166 11158612 PMC14726

[174] Casellas R Basu U Yewdell WT Chaudhuri J Robbiani DF Di Noia JM. Mutations, kataegis and translocations in B cells: understanding AID promiscuous activity. Nat Rev Immunol (2016) 16(3):164–76. 10.1038/nri.2016.2 26898111 PMC4871114

[175] Scheeren FA Nagasawa M Weijer K Cupedo T Kirberg J Legrand N T cell-independent development and induction of somatic hypermutation in human IgM+ IgD+ CD27+ B cells. J Exp Med (2008) 205(9):2033–42. 10.1084/jem.20070447 18695003 PMC2526198

[176] Muzio M Apollonio B Scielzo C Frenquelli M Vandoni I Boussiotis V Constitutive activation of distinct BCR-signaling pathways in a subset of CLL patients: a molecular signature of anergy. Blood (2008) 112(1):188–95. 10.1182/blood-2007-09-111344 18292287

[177] Mockridge CI Potter KN Wheatley I Neville LA Packham G Stevenson FK. Reversible anergy of sIgM-mediated signaling in the two subsets of CLL defined by VH-gene mutational status. Blood (2007) 109(10):4424–31. 10.1182/blood-2006-11-056648 17255355

[178] Guo B Zhang L Chiorazzi N Rothstein TL. IL-4 rescues surface IgM expression in chronic lymphocytic leukemia. Blood (2016) 128(4):553–62. 10.1182/blood-2015-11-682997 27226435 PMC4965907

[179] Fais F Sellars B Ghiotto F Yan XJ Dono M Allen SL Examples of in vivo isotype class switching in IgM+ chronic lymphocytic leukemia B cells. J Clin Invest (1996) 98(7):1659–66. 10.1172/JCI118961 8833916 PMC507600

[180] Oppezzo P Vuillier F Vasconcelos Y Dumas G Magnac C Payelle-Brogard B Chronic lymphocytic leukemia B cells expressing AID display dissociation between class switch recombination and somatic hypermutation. Blood (2003) 101(10):4029–32. 10.1182/blood-2002-10-3175 12521993

[181] Malisan F Fluckiger AC Ho S Guret C Banchereau J Martinez-Valdez H. B-chronic lymphocytic leukemias can undergo isotype switching in vivo and can be induced to differentiate and switch in vitro. Blood (1996) 87(2):717–24. 8555496

[182] Palacios F Moreno P Morande P Abreu C Correa A Porro V High expression of AID and active class switch recombination might account for a more aggressive disease in unmutated CLL patients: link with an activated microenvironment in CLL disease. Blood (2010) 115(22):4488–96. 10.1182/blood-2009-12-257758 20233972

[183] Efremov DG Ivanovski M Batista FD Pozzato G Burrone OR. IgM-producing chronic lymphocytic leukemia cells undergo immunoglobulin isotype-switching without acquiring somatic mutations. J Clin Invest (1996) 98(2):290–8. 10.1172/JCI118792 8755637 PMC507430

[184] Cerutti A Zan H Kim EC Shah S Schattner EJ Schaffer A Ongoing in vivo immunoglobulin class switch DNA recombination in chronic lymphocytic leukemia B cells. J Immunol (2002) 169(11):6594–603. 10.4049/jimmunol.169.11.6594 12444172 PMC4625981

[185] Gurrieri C McGuire P Zan H Yan XJ Cerutti A Albesiano E Chronic lymphocytic leukemia B cells can undergo somatic hypermutation and intraclonal immunoglobulin V(H)DJ(H) gene diversification. J Exp Med (2002) 196(5):629–39. 10.1084/jem.20011693 12208878 PMC2194006

[186] Sutton LA Kostareli E Hadzidimitriou A Darzentas N Tsaftaris A Anagnostopoulos A Extensive intraclonal diversification in a subgroup of chronic lymphocytic leukemia patients with stereotyped IGHV4-34 receptors: implications for ongoing interactions with antigen. Blood (2009) 114(20):4460–8. 10.1182/blood-2009-05-221309 19713457

[187] Stamatopoulos B Timbs A Bruce D Smith T Clifford R Robbe P Targeted deep sequencing reveals clinically relevant subclonal IgHV rearrangements in chronic lymphocytic leukemia. Leukemia (2017) 31(4):837–45. 10.1038/leu.2016.307 27795555

[188] Kriangkum J Motz SN Mack T Beiggi S Baigorri E Kuppusamy H Single-cell analysis and next-generation immuno-sequencing show that multiple clones persist in patients with chronic lymphocytic leukemia. PLoS One (2015) 10(9):e0137232. 10.1371/journal.pone.0137232 26353109 PMC4564241

[189] Messmer BT Albesiano E Messmer D Chiorazzi N. The pattern and distribution of immunoglobulin VH gene mutations in chronic lymphocytic leukemia B cells are consistent with the canonical somatic hypermutation process. Blood (2004) 103(9):3490–5. 10.1182/blood-2003-10-3407 14695232

[190] Juliusson G Robert KH Hammarstrom L Smith CI Biberfeld G Gahrton G. Mitogen-induced switching of immunoglobulin heavy-chain class secretion in chronic B-lymphocytic leukaemia and immunocytoma cell populations. Scand J Immunol (1983) 17(1):51–9. 10.1111/j.1365-3083.1983.tb00765.x 6405478

[191] Steinberg J Moore MA Bernhardt B Bona CA Platsoucas CD. Induction of proliferation and differentiation of leukaemic B cells from patients with chronic lymphocytic leukaemia by anti-mu and conditioned medium. Scand J Immunol (1987) 25(6):599–611. 10.1111/j.1365-3083.1987.tb01086.x 3110941

[192] Fluckiger AC Rossi JF Bussel A Bryon P Banchereau J Defrance T. Responsiveness of chronic lymphocytic leukemia B cells activated via surface Igs or CD40 to B-cell tropic factors. Blood (1992) 80(12):3173–81. 1281692

[193] Robert KH Bird AG Moller E. Mitogen-induced differentiation of human CLL lymphocytes to antibody-secreting cells. Scand J Immunol (1979) 10(5):447–52. 10.1111/j.1365-3083.1979.tb01374.x 232570

[194] Tangye SG Weston KM Raison RL. Phorbol ester activates CD5+ leukaemic B cells via a T cell-independent mechanism. Immunol Cell Biol (1995) 73(1):44–51. 10.1038/icb.1995.7 7539402

[195] van Kooten C Rensink I Aarden L van Oers R. Effect of IL-4 and IL-6 on the proliferation and differentiation of B-chronic lymphocytic leukemia cells. Leukemia (1993) 7(4):618–24. 8464239

[196] Ghamlouch H Ouled-Haddou H Guyart A Regnier A Trudel S Claisse JF Phorbol myristate acetate, but not CD40L, induces the differentiation of CLL B cells into Ab-secreting cells. Immunol Cell Biol (2014) 92(7):591–604. 10.1038/icb.2014.37 24797583 PMC4134517

[197] Wakai M Hashimoto S Omata M Sthoeger ZM Allen SL Lichtman SM IgG+, CD5+ human chronic lymphocytic leukemia B cells. Production of IgG antibodies that exhibit diminished autoreactivity and IgG subclass skewing. Autoimmunity (1994) 19(1):39–48. 10.3109/08916939409008007 7538331

[198] Matolcsy A Casali P Nador RG Liu YF Knowles DM. Molecular characterization of IgA- and/or IgG-switched chronic lymphocytic leukemia B cells. Blood (1997) 89(5):1732–9. 9057657 PMC4631049

[199] Geisler CH Larsen JK Hansen NE Hansen MM Christensen BE Lund B Prognostic importance of flow cytometric immunophenotyping of 540 consecutive patients with B-cell chronic lymphocytic leukemia. Blood (1991) 78(7):1795–802. 1717071

[200] Vardi A Agathangelidis A Sutton LA Chatzouli M Scarfo L Mansouri L IgG-switched CLL has a distinct immunogenetic signature from the common MD variant: ontogenetic implications. Clin Cancer Res (2014) 20(2):323–30. 10.1158/1078-0432.CCR-13-1993 24240110

[201] Hashimoto S Dono M Wakai M Allen SL Lichtman SM Schulman P Somatic diversification and selection of immunoglobulin heavy and light chain variable region genes in IgG+ CD5+ chronic lymphocytic leukemia B cells. J Exp Med (1995) 181(4):1507–17. 10.1084/jem.181.4.1507 7535340 PMC2191964

[202] Murray F Darzentas N Hadzidimitriou A Tobin G Boudjogra M Scielzo C Stereotyped patterns of somatic hypermutation in subsets of patients with chronic lymphocytic leukemia: implications for the role of antigen selection in leukemogenesis. Blood (2008) 111(3):1524–33. 10.1182/blood-2007-07-099564 17959859

[203] Vardi A Agathangelidis A Sutton LA Ghia P Rosenquist R Stamatopoulos K. Immunogenetic studies of chronic lymphocytic leukemia: revelations and speculations about ontogeny and clinical evolution. Cancer Res (2014) 74(16):4211–6. 10.1158/0008-5472.CAN-14-0630 25074616

[204] Martin V Wu YC Kipling D Dunn-Walters DK. Age-related aspects of human IgM(+) B cell heterogeneity. Ann N Y Acad Sci (2015) 1362:153–63. 10.1111/nyas.12823 26152370 PMC4758400

[205] Wu YC Kipling D Leong HS Martin V Ademokun AA Dunn-Walters DK. High-throughput immunoglobulin repertoire analysis distinguishes between human IgM memory and switched memory B-cell populations. Blood (2010) 116(7):1070–8. 10.1182/blood-2010-03-275859 20457872 PMC2938129

[206] Forconi F Potter KN Wheatley I Darzentas N Sozzi E Stamatopoulos K The normal IGHV1-69-derived B-cell repertoire contains stereotypic patterns characteristic of unmutated CLL. Blood (2010) 115(1):71–7. 10.1182/blood-2009-06-225813 19887677

[207] Plevova K Francova HS Burckova K Brychtova Y Doubek M Pavlova S Multiple productive immunoglobulin heavy chain gene rearrangements in chronic lymphocytic leukemia are mostly derived from independent clones. Haematologica (2014) 99(2):329–38. 10.3324/haematol.2013.087593 24038023 PMC3912964

[208] Heyman B Volkheimer AD Weinberg JB Double IGHV DNA gene rearrangements in CLL: influence of mixed-mutated and -unmutated rearrangements on outcomes in CLL. Blood Cancer J (2016) 6(7):e440 10.1038/bcj.2016.49 27367477 PMC5030375

[209] Klinger M Zheng J Elenitoba-Johnson KS Perkins SL Faham M Bahler DW. Next-generation IgVH sequencing CLL-like monoclonal B-cell lymphocytosis reveals frequent oligoclonality and ongoing hypermutation. Leukemia (2016) 30(5):1055–61. 10.1038/leu.2015.351 26686246

[210] Marcatili P Ghiotto F Tenca C Chailyan A Mazzarello AN Yan XJ Igs expressed by chronic lymphocytic leukemia B cells show limited binding-site structure variability. J Immunol (2013) 190(11):5771–8. 10.4049/jimmunol.1300321 23636053

[211] Herve M Xu K Ng YS Wardemann H Albesiano E Messmer BT Unmutated and mutated chronic lymphocytic leukemias derive from self-reactive B cell precursors despite expressing different antibody reactivity. J Clin Invest (2005) 115(6):1636–43. 10.1172/JCI24387 15902303 PMC1088018

[212] Lanemo Myhrinder A Hellqvist E Sidorova E Soderberg A Baxendale H Dahle C A new perspective: molecular motifs on oxidized LDL, apoptotic cells, and bacteria are targets for chronic lymphocytic leukemia antibodies. Blood (2008) 111(7):3838–48. 10.1182/blood-2007-11-125450 18223168

[213] Holodick NE Rodriguez-Zhurbenko N Hernandez AM. Defining natural antibodies. Front Immunol (2017) 8:872. 10.3389/fimmu.2017.00872 28798747 PMC5526850

[214] Sthoeger ZM Wakai M Tse DB Vinciguerra VP Allen SL Budman DR Production of autoantibodies by CD5-expressing B lymphocytes from patients with chronic lymphocytic leukemia. J Exp Med (1989) 169(1):255–68. 10.1084/jem.169.1.255 2462608 PMC2189197

[215] Borche L Lim A Binet JL Dighiero G. Evidence that chronic lymphocytic leukemia B lymphocytes are frequently committed to production of natural autoantibodies. Blood (1990) 76(3):562–9. 2378986

[216] Chu CC Catera R Hatzi K Yan XJ Zhang L Wang XB Chronic lymphocytic leukemia antibodies with a common stereotypic rearrangement recognize nonmuscle myosin heavy chain IIA. Blood (2008) 112(13):5122–9. 10.1182/blood-2008-06-162024 18812466 PMC2597608

[217] Binder M Muller F Frick M Wehr C Simon F Leistler B CLL B-cell receptors can recognize themselves: alternative epitopes and structural clues for autostimulatory mechanisms in CLL. Blood (2013) 121(1):239–41. 10.1182/blood-2012-09-454439 23287626

[218] Hoogeboom R van Kessel KP Hochstenbach F Wormhoudt TA Reinten RJ Wagner K A mutated B cell chronic lymphocytic leukemia subset that recognizes and responds to fungi. J Exp Med (2013) 210(1):59–70. 10.1084/jem.20121801 23296468 PMC3549718

[219] Hwang KK Trama AM Kozink DM Chen X Wiehe K Cooper AJ IGHV1-69 B cell chronic lymphocytic leukemia antibodies cross-react with HIV-1 and hepatitis C virus antigens as well as intestinal commensal bacteria. PLoS One (2014) 9(3):e90725. 10.1371/journal.pone.0090725 24614505 PMC3948690

[220] Bomben R Dal-Bo M Benedetti D Capello D Forconi F Marconi D Expression of mutated IGHV3-23 genes in chronic lymphocytic leukemia identifies a disease subset with peculiar clinical and biological features. Clin Cancer Res (2010) 16(2):620–8. 10.1158/1078-0432.CCR-09-1638 20068100

[221] Hatzi K Catera R Moreno Atanasio C Fischetti VA Allen SL Kolitz JE Chronic lymphocytic leukemia immunoglobulins display bacterial reactivity that converges and diverges from auto-/poly-reactivity and IGHV mutation status. Clin Immunol (2016) 172:44–51. 10.1016/j.clim.2016.08.020 27586592

[222] Cantaert T Schickel JN Bannock JM Ng YS Massad C Delmotte FR Decreased somatic hypermutation induces an impaired peripheral B cell tolerance checkpoint. J Clin Invest (2016) 126(11):4289–302. 10.1172/JCI84645 27701145 PMC5096912

[223] Patten PE Chu CC Albesiano E Damle RN Yan XJ Kim D IGHV-unmutated and IGHV-mutated chronic lymphocytic leukemia cells produce activation-induced deaminase protein with a full range of biologic functions. Blood (2012) 120(24):4802–11. 10.1182/blood-2012-08-449744 23071276 PMC3520620

[224] Steinberg J Bona C Polyzos A Moore MA Platsoucas CD. Proliferation of leukemic B cells in response to SAC and anti-mu. Evidence for different modes of action and comparison to proliferation and differentiation induced by conditioned medium. Leuk Res (1988) 12(7):559–66. 10.1016/0145-2126(88)90085-9 3262791

[225] Oppenheim JJ Whang J Frei E III Immunologic and cytogenetic studies of chronic lymphocytic leukemic cells. Blood (1965) 26:121–32. 14332475

[226] Hanley-Hyde JM Lynch RG. The physiology of B cells as studied with tumor models. Annu Rev Immunol (1986) 4:621–49. 10.1146/annurev.iy.04.040186.003201 3518751

[227] Gordon J Mellstedt H Aman P Biberfeld P Bjorkholm M Klein G. Phenotypes in chronic B-lymphocytic leukemia probed by monoclonal antibodies and immunoglobulin secretion studies: identification of stages of maturation arrest and the relation to clinical findings. Blood (1983) 62(4):910–7. 6603884

[228] Kishimoto T. Human neoplastic B cells: monoclonal models of B-cell differentiation. Immunol Today (1983) 4(4):117–20. 10.1016/0167-5699(83)90021-X 25291478

[229] Galton DA. The pathogenesis of chronic lymphocytic leukemia. Can Med Assoc J (1966) 94(19):1005–10. 4952384 PMC1936613

[230] Cohnen G Douglas SD Konig E Brittinger G Pokeweed mitogen response of lymphocytes in chronic lymphocytes in chronic lymphocytic leukemia: a fine structural study. Blood (1973) 42(4):591–600. 4795012

[231] Alley CL Wang E Dunphy CH Gong JZ Lu CM Boswell EL Diagnostic and clinical considerations in concomitant bone marrow involvement by plasma cell myeloma and chronic lymphocytic leukemia/monoclonal B-cell lymphocytosis: a series of 15 cases and review of literature. Arch Pathol Lab Med (2013) 137(4):503–17. 10.5858/arpa.2011-0696-OA 23544940

[232] Trudel S Ghamlouch H Dremaux J Delette C Harrivel V Marolleau JP The importance of an in-depth study of immunoglobulin gene rearrangements when ascertaining the clonal relationship between concomitant chronic lymphocytic leukemia and multiple myeloma. Front Immunol (2016) 7:625. 10.3389/fimmu.2016.00625 28082975 PMC5187371

[233] Rubartelli A Sitia R Zicca A Grossi CE Ferrarini M. Differentiation of chronic lymphocytic leukemia cells: correlation between the synthesis and secretion of immunoglobulins and the ultrastructure of the malignant cells. Blood (1983) 62(2):495–504. 6603243

[234] Saltman DL Ross JA Banks RE Ross FM Ford AM Mackie MJ. Molecular evidence for a single clonal origin in biphenotypic concomitant chronic lymphocytic leukemia and multiple myeloma. Blood (1989) 74(6):2062–5. 2804347

[235] Fermand JP James JM Herait P Brouet JC. Associated chronic lymphocytic leukemia and multiple myeloma: origin from a single clone. Blood (1985) 66(2):291–3. 2990608

[236] Murphy JJ Norton JD. Phorbol ester induction of early response gene expression in lymphocytic leukemia and normal human B-cells. Leuk Res (1993) 17(8):657–62. 10.1016/0145-2126(93)90070-2 8355509

[237] Segel GB Woodlock TJ Xu J Li L Felgar RE Ryan DH Early gene activation in chronic leukemic B lymphocytes induced toward a plasma cell phenotype. Blood Cells Mol Dis (2003) 30(3):277–87. 10.1016/S1079-9796(03)00035-4 12737946

[238] Gignac SM Buschle M Hoffbrand AV Drexler HG. Down-regulation of CD5 mRNA in B-chronic lymphocytic leukemia cells by differentiation-inducing agents. Eur J Immunol (1990) 20(5):1119–23. 10.1002/eji.1830200526 1694132

[239] Gordon J Mellstedt H Aman P Biberfeld P Klein G. Phenotypic modulation of chronic lymphocytic leukemia cells by phorbol ester: induction of IgM secretion and changes in the expression of B cell-associated surface antigens. J Immunol (1984) 132(1):541–7. 6606672

[240] Carlsson M Soderberg O Nilsson K. Interleukin-4 (IL-4) enhances homotypic adhesion of activated B-chronic lymphocytic leukaemia (B-CLL) cells via a selective up-regulation of CD54. Scand J Immunol (1993) 37(4):515–22. 10.1111/j.1365-3083.1993.tb03328.x 8097058

[241] Okamura J Letarte M Stein LD Sigal NH Gelfand EW. Modulation of chronic lymphocytic leukemia cells by phorbol ester: increase in Ia expression, IgM secretion and MLR stimulatory capacity. J Immunol (1982) 128(5):2276–80. 6460820

[242] Polliack A. 12-0-tetradecanoyl phorbol-13-acetate (TPA) and its effect on leukaemic cells, in-vitro-A review. Leuk Lymphoma (1990) 3(3):173–82. 10.3109/10428199009050993 27457435

[243] Kazi JU Kabir NN Ronnstrand L. Protein kinase C (PKC) as a drug target in chronic lymphocytic leukemia. Med Oncol (2013) 30(4):757. 10.1007/s12032-013-0757-7 24174318

[244] Carlsson M Totterman TH Matsson P Nilsson K. Cell cycle progression of B-chronic lymphocytic leukemia cells induced to differentiate by TPA. Blood (1988) 71(2):415–21. 3257396

[245] Carlsson M Matsson P Rosen A Sundstrom C Totterman TH Nilsson K. Phorbol ester and B cell-stimulatory factor synergize to induce B-chronic lymphocytic leukemia cells to simultaneous immunoglobulin secretion and DNA synthesis. Leukemia (1988) 2(11):734–44. 3263557

[246] Totterman TH Nilsson K Sundstrom C. Phorbol ester-induced differentiation of chronic lymphocytic leukaemia cells. Nature (1980) 288(5787):176–8. 10.1038/288176a0 7432517

[247] Engel P Ingles J de la Calle O Gallart T. Cellular activation without proliferation to B cell growth factor and interleukin 2 in chronic lymphocytic leukaemia B cells stimulated with phorbol ester plus calcium ionophore. Clin Exp Immunol (1989) 76(1):61–7. 2500274 PMC1541746

[248] Roifman CM Benedict SH Cheung RK Gelfand EW. Induction of human B cell proliferation and differentiation by the combination of phorbol ester and ionomycin. Eur J Immunol (1987) 17(5):701–6. 10.1002/eji.1830170519 3495445

[249] Ingles J Engel P De La Calle O Gallart T. Differential responsiveness of human B lymphocytes to phorbol ester and calcium ionophore based on their state of activation. Immunology (1989) 67(3):359–64. 2503437 PMC1385353

[250] Clevers HC Versteegen JM Logtenberg T Gmelig-Meyling FH Ballieux RE. Synergistic action of A23187 and phorbol ester on human B cell activation. J Immunol (1985) 135(6):3827–30. 3934268

[251] Franz A Bryant A Farrant J. Interleukin-2-induced DNA synthesis and immunoglobulin secretion by resting human tonsillar B cells: effects of protein kinase C activation. Immunology (1991) 73(3):322–6. 1879879 PMC1384550

[252] Freedman AS Boyd AW Bieber FR Daley J Rosen K Horowitz JC Normal cellular counterparts of B cell chronic lymphocytic leukemia. Blood (1987) 70(2):418–27. 3496927

[253] Zupo S Dono M Massara R Taborelli G Chiorazzi N Ferrarini M. Expression of CD5 and CD38 by human CD5- B cells: requirement for special stimuli. Eur J Immunol (1994) 24(6):1426–33. 10.1002/eji.1830240628 7515814

[254] Youinou P Mackenzie L Jouquan J Le Goff P Lydyard PM. CD5 positive B cells in patients with rheumatoid arthritis: phorbol ester mediated enhancement of detection. Ann Rheum Dis (1987) 46(1):17–22. 10.1136/ard.46.1.17 3101622 PMC1002052

[255] Grandjenette C Kennel A Faure GC Bene MC Feugier P. Expression of functional toll-like receptors by B-chronic lymphocytic leukemia cells. Haematologica (2007) 92(9):1279–81. 10.3324/haematol.10975 17768129

[256] Liang X Moseman EA Farrar MA Bachanova V Weisdorf DJ Blazar BR Toll-like receptor 9 signaling by CpG-B oligodeoxynucleotides induces an apoptotic pathway in human chronic lymphocytic leukemia B cells. Blood (2010) 115(24):5041–52. 10.1182/blood-2009-03-213363 20339095 PMC2890142

[257] Mongini PK Gupta R Boyle E Nieto J Lee H Stein J TLR-9 and IL-15 synergy promotes the in vitro clonal expansion of chronic lymphocytic leukemia B cells. J Immunol (2015) 195(3):901–23. 10.4049/jimmunol.1403189 26136429 PMC4505957

[258] Decker T Schneller F Hipp S Miething C Jahn T Duyster J Cell cycle progression of chronic lymphocytic leukemia cells is controlled by cyclin D2, cyclin D3, cyclin-dependent kinase (cdk) 4 and the cdk inhibitor p27. Leukemia (2002) 16(3):327–34. 10.1038/sj.leu.2402389 11896535

[259] Nolz JC Tschumper RC Pittner BT Darce JR Kay NE Jelinek DF. ZAP-70 is expressed by a subset of normal human B-lymphocytes displaying an activated phenotype. Leukemia (2005) 19(6):1018–24. 10.1038/sj.leu.2403726 15800671

[260] Burgler S Gimeno A Parente-Ribes A Wang D Os A Devereux S Chronic lymphocytic leukemia cells express CD38 in response to Th1 cell-derived IFN-gamma by a T-bet-dependent mechanism. J Immunol (2015) 194(2):827–35. 10.4049/jimmunol.1401350 25505279

[261] Os A Burgler S Ribes AP Funderud A Wang D Thompson KM Chronic lymphocytic leukemia cells are activated and proliferate in response to specific T helper cells. Cell Rep (2013) 4(3):566–77. 10.1016/j.celrep.2013.07.011 23933259

[262] Patten PE Buggins AG Richards J Wotherspoon A Salisbury J Mufti GJ CD38 expression in chronic lymphocytic leukemia is regulated by the tumor microenvironment. Blood (2008) 111(10):5173–81. 10.1182/blood-2007-08-108605 18326821

[263] Ghia P Strola G Granziero L Geuna M Guida G Sallusto F Chronic lymphocytic leukemia B cells are endowed with the capacity to attract CD4+, CD40L+ T cells by producing CCL22. Eur J Immunol (2002) 32(5):1403–13. 10.1002/1521-4141(200205)32:5<1403::AID-IMMU1403>3.0.CO;2-Y 11981828

[264] Fluckiger AC Garrone P Durand I Galizzi JP Banchereau J. Interleukin 10 (IL-10) upregulates functional high affinity IL-2 receptors on normal and leukemic B lymphocytes. J Exp Med (1993) 178(5):1473–81. 10.1084/jem.178.5.1473 8228801 PMC2191252

[265] Plander M Seegers S Ugocsai P Diermeier-Daucher S Ivanyi J Schmitz G Different proliferative and survival capacity of CLL-cells in a newly established in vitro model for pseudofollicles. Leukemia (2009) 23(11):2118–28. 10.1038/leu.2009.145 19657365

[266] Van den Hove LE Van Gool SW Vandenberghe P Bakkus M Thielemans K Boogaerts MA CD40 triggering of chronic lymphocytic leukemia B cells results in efficient alloantigen presentation and cytotoxic T lymphocyte induction by up-regulation of CD80 and CD86 costimulatory molecules. Leukemia (1997) 11(4):572–80. 10.1038/sj.leu.2400598 9096698

[267] Ghamlouch H Ouled-Haddou H Damaj G Royer B Gubler B Marolleau JP. A combination of cytokines rescues highly purified leukemic CLL B-cells from spontaneous apoptosis in vitro. PLoS One (2013) 8(3):e60370. 10.1371/journal.pone.0060370 23555960 PMC3608602

[268] Aguilar-Hernandez MM Blunt MD Dobson R Yeomans A Thirdborough S Larrayoz M IL-4 enhances expression and function of surface IgM in CLL cells. Blood (2016) 127(24):3015–25. 10.1182/blood-2015-11-682906 27002119

[269] Gitelson E Hammond C Mena J Lorenzo M Buckstein R Berinstein NL Chronic lymphocytic leukemia-reactive T cells during disease progression and after autologous tumor cell vaccines. Clin Cancer Res (2003) 9(5):1656–65. 12738718

[270] Dorfman DM Hwang ES Shahsafaei A Glimcher LH. T-bet, a T-cell-associated transcription factor, is expressed in a subset of B-cell lymphoproliferative disorders. Am J Clin Pathol (2004) 122(2):292–7. 10.1309/AQQ2DVM75DVY0PWP 15323146

[271] Strati P Shanafelt TD. Monoclonal B-cell lymphocytosis and early-stage chronic lymphocytic leukemia: diagnosis, natural history, and risk stratification. Blood (2015) 126(4):454–62. 10.1182/blood-2015-02-585059 26065657 PMC4624440

[272] Landgren O Albitar M Ma W Abbasi F Hayes RB Ghia P B-cell clones as early markers for chronic lymphocytic leukemia. N Engl J Med (2009) 360(7):659–67. 10.1056/NEJMoa0806122 19213679 PMC7015348

[273] Lanasa MC Allgood SD Volkheimer AD Gockerman JP Whitesides JF Goodman BK Single-cell analysis reveals oligoclonality among ‘low-count’ monoclonal B-cell lymphocytosis. Leukemia (2010) 24(1):133–40. 10.1038/leu.2009.192 19946263 PMC2806490

[274] Kikushige Y Ishikawa F Miyamoto T Shima T Urata S Yoshimoto G Self-renewing hematopoietic stem cell is the primary target in pathogenesis of human chronic lymphocytic leukemia. Cancer Cell (2011) 20(2):246–59. 10.1016/j.ccr.2011.06.029 21840488

[275] Damm F Mylonas E Cosson A Yoshida K Della Valle V Mouly E Acquired initiating mutations in early hematopoietic cells of CLL patients. Cancer Discov (2014) 4(9):1088–101. 10.1158/2159-8290.CD-14-0104 24920063

[276] Montecino-Rodriguez E Berent-Maoz B Dorshkind K. Causes, consequences, and reversal of immune system aging. J Clin Invest (2013) 123(3):958–65. 10.1172/JCI64096 23454758 PMC3582124

[277] Shlush LI Zandi S Itzkovitz S Schuh AC. Aging, clonal hematopoiesis and preleukemia: not just bad luck? Int J Hematol (2015) 102(5):513–22. 10.1007/s12185-015-1870-5 26440972

[278] Dunn-Walters DK. The ageing human B cell repertoire: a failure of selection? Clin Exp Immunol (2016) 183(1):50–6. 10.1111/cei.12700 26332693 PMC4687518

[279] DiLillo DJ Weinberg JB Yoshizaki A Horikawa M Bryant JM Iwata Y Chronic lymphocytic leukemia and regulatory B cells share IL-10 competence and immunosuppressive function. Leukemia (2013) 27(1):170–82. 10.1038/leu.2012.165 22713648 PMC3742013

